# Functional feeds marginally alter immune expression and microbiota of Atlantic salmon (*Salmo salar*) gut, gill, and skin mucosa though evidence of tissue-specific signatures and host–microbe coadaptation remain

**DOI:** 10.1186/s42523-022-00173-0

**Published:** 2022-03-10

**Authors:** Jacob W. Bledsoe, Michael R. Pietrak, Gary S. Burr, Brian C. Peterson, Brian C. Small

**Affiliations:** 1 Hagerman Fish Culture Experiment Station, Aquaculture Research Institute, University of Idaho, 3059-F National Fish Hatchery Rd., Hagerman, ID 83332 USA; 2 Agricultural Research Service, National Cold Water Marine Aquaculture Center, United States Department of Agriculture, 25 Salmon Farm Road, Franklin, ME 04634 USA

**Keywords:** Host–microbiota interactions, Functional feeds, Mannan-oligosaccharides, Coconut oil, Immune regulation, Gene expression, Fish microbiome, Atlantic salmon, Sea lice

## Abstract

**Background:**

Mucosal surfaces of fish provide cardinal defense against environmental pathogens and toxins, yet these external mucosae are also responsible for maintaining and regulating beneficial microbiota. To better our understanding of interactions between host, diet, and microbiota in finfish and how those interactions may vary across mucosal tissue, we used an integrative approach to characterize and compare immune biomarkers and microbiota across three mucosal tissues (skin, gill, and gut) in Atlantic salmon receiving a control diet or diets supplemented with mannan-oligosaccharides, coconut oil, or both. Dietary impacts on mucosal immunity were further evaluated by experimental ectoparasitic sea lice (*Lepeophtheirus salmonis*) challenge.

**Results:**

Fish grew to a final size of 646.5 g ± 35.8 during the 12-week trial, with no dietary effects on growth or sea lice resistance. Bacterial richness differed among the three tissues with the highest richness detected in the gill, followed by skin, then gut, although dietary effects on richness were only detected within skin and gill. Shannon diversity was reduced in the gut compared to skin and gill but was not influenced by diet. Microbiota communities clustered separately by tissue, with dietary impacts on phylogenetic composition only detected in the skin, although skin and gill communities showed greater overlap compared to the gut according to overall composition, differential abundance, and covariance networks. Inferred metagenomic functions revealed preliminary evidence for tissue-specific host–microbiota coadaptation, as putative microbiota functions showed ties to the physiology of each tissue. Immune gene expression profiles displayed tissue-specific signatures, yet dietary effects were also detected within each tissue and peripheral blood leukocytes. Procrustes analysis comparing sample-matched multivariate variation in microbiota composition to that of immune expression profiles indicated a highly significant correlation between datasets.

**Conclusions:**

Diets supplemented with functional ingredients, namely mannan-oligosaccharide, coconut oil, or a both, resulted in no difference in Atlantic salmon growth or resistance to sea lice infection. However, at the molecular level, functional ingredients caused physiologically relevant changes to mucosal microbiota and host immune expression. Putative tissue-specific metagenomic functions and the high correlation between expression profiles and microbiota composition suggest host and microbiota are interdependent and coadapted in a tissue-specific manner.

**Supplementary Information:**

The online version contains supplementary material available at 10.1186/s42523-022-00173-0.

## Background

Mucosal surfaces lie at the interface between organism and environment and serve as the first line of defense against pathogens, pollutants, and other stressors. The importance of mucosal tissues has generated substantial research interest and concurrent scientific discovery related to teleost mucosal immunity over the last two decades. We now know the complexity of the systemic immune system pales in comparison to mucosal immune systems, which can be subdivided into distinct mucosa-associated lymphoid tissues (MALT). Generally, among teleost fish the MALT includes the gut-associated lymphoid tissue (GALT), skin-associated lymphoid tissue (SALT) and gill-associated lymphoid tissue (GiALT) [71]. These MALT represent tissue-specific centers from which the immune system regulates environmental microorganisms through both innate and adaptive immune effectors. The innate arm of mucosal immunity is the first response against microbial invasion, particularly in fish. Innate mucosal immunity includes host secretions that directly interact with microbes such as mucins, antimicrobial peptides, enzymes, and complement components,phagocytic cells that engulf pathogens; as well as pathogen recognition receptors (PRR) and local cytokine signaling that regulates local inflammation [28]. Mucosal adaptive immune responses are primarily achieved by secreted or membrane bound mucosal immunoglobulins (IgT/IgM) generated by local B-cells and plasmoblasts, as well as CD4 + (T-helper − T_H_), CD8 + (cytotoxic-T − T_C_), and FOXP3 + (regulatory-T − T_Reg_) T-cells [69]. The adaptive immune system further relies on cell-to-cell communication through the major-histocompatibility complexes (MHC-I and MHC-II) to regulate and signal these adaptive responses. Together, these systems are responsible for providing an immunological barrier against pathogens from the external environment, which is particularly important in aquatic animals due to the intimate interaction with environmental microbes [16, 69].

Mucosal immune tissues of fish are not only responsible for preventing pathogenic invasion but must also interact with and attempt to govern the microbiota, or commensal and beneficial microbes that continuously inhabit all mucosal surfaces. Research on the mucosal microbiota of fish has flourished in the last decade and expanded our understanding of the importance and diversity of physiological impacts that mucosal microbes have on their host. Evidence from axenic and gnotobiotic zebrafish models suggest microbiota serve a critical role in priming and maintaining the development and activity of the teleost immune system [71]. To date, most research on fish mucosal microbiota has been focused on gut microbiota, with the gut microbes of over 150 teleost species characterized by next- generation sequencing, across a range of environmental conditions [60]. Despite early focus on gut microbiota, the skin, gill, and even nasopharyngeal microbiomes of fish are now receiving more attention, though the functional attributes of these microbiota and their interactions with the host remain greatly understudied [44].

As the first line of immunological defense, it is not surprising that many of the most financially burdensome diseases affecting aquaculture production begin as acute perturbations to one or more of the mucosal tissues. For example, commercial aquaculture must push towards increasing amounts of terrestrial plant-based ingredients in diets to replace fishmeal and fish oil for industry growth to remain financially and environmentally feasible [57],however, at high levels these ingredients induce inflammatory enteritis in the gut mucosa and dysregulate gut microbiota, particularly in high-value carnivorous finfish [38]. In addition, pathogens, including those which cause infectious salmon anemia, enteric red mouth (i.e., yersiniosis), amoebic gill disease, and white-spot disease (Ichthyophthiriasis), are all known to initiate virulence at the site of the gill mucosa [39]. Furthermore, ectoparasitic infections such as those from sea lice (*Lepeophtherius* and *Caligus*), which represent the largest disease-related production-cost impacting Atlantic salmon aquaculture [6], as well as *Ichthyophthirius* and numerous bacterial pathogens (i.e., *Aeromonas*, *Flavobacterium*, and *Vibrio*) are known to afflict the skin mucosa of aquaculture finfish [2]. Improving our understanding of finfish mucosal health and the interaction with microbiota has the potential to not only increase our ability to better manage such disease outbreaks but may also help prevent disease in some instances by improving our ability to generate practical and efficacious supplements or mucosal vaccinations that can be easily delivered orally or via bath immersion [1].

Functional feed ingredients are commonly tested in aquaculture as a means of improving mucosal health in fish. In the context of aquaculture, functional feeds are defined as dietary supplements that enhance growth, health, and physiological performance when administered above basal dietary requirements and can include micro-nutrients, immune stimulants, specific lipid sources, or pre-, pro-, and synbiotics [50]. Mannan-oligosaccharides (MOS), complex carbohydrate molecules derived from yeast cell walls, are commonly used as functional ingredients, and are thought to serve both prebiotic and immune stimulant functions. Known benefits of MOS supplementation include superior histomorphology following dietary or pathogenic perturbation of the intestine [76–78, 30,43], skin [43] or gill [84], increased production and altered proteome of skin mucus [68, 53], and modulation of gut microbiota [18, 19]. Mechanisms involved in these outcomes are thought to be predominantly based in the ability of MOS molecules to (1) stimulate PRR leading to downstream alterations in local and systemic immunity, (2) bind to and neutralize some enteric pathogens, and (3) serve as a preferred fermentable prebiotic carbohydrate to nourish specific microbiota [43].

Specific oil sources have also received attention as functional ingredients in aquaculture. Differences in fatty acid profiles among various dietary lipid sources are known to influence (1) cell membrane structure, function, and fluidity, (2) the production of immunologically active eicosanoids (i.e., prostaglandins, thromboxanes, and leukotrienes), (3) oxidative stress, and (4) energy metabolism [75]. Oil sources high in medium-chain fatty-acids (MCFA), those with a chain length of 6–12 carbons, have also received interest as novel dietary energy sources because of the ease with which MCFA can undergo beta-oxidation to produce energy [48], while some MCFA have also been shown to have more functional attributes as well. Coconut oil is a lipid source with high levels of saturated MCFA, particularly lauric (C12:0,40–50% and caprylic acid (C8:0; 5–10%. In-vitro assessment of lauric and caprylic acid have shown them to have antimicrobial [35] and antiparasitic properties [32], respectively. Nevertheless, the in-vivo effects of high levels of dietary coconut oil and its associated MCFA profile on fish mucosal health have yet to be fully explored.

In what follows, an integrative approach was taken to compare host-microbiota interactions across the skin, gut, and gill microbiota of Atlantic salmon (Salmo salar) while also exploring the concurrent impacts of diets supplemented with functional ingredients: (1) a control (Control), (2) 1% (10 g kg-1) mannan oligosaccharides supplementation (MOS), (3) a 96% lipid replacement with coconut oil (CoconutOil), or (4) a combination of the two (CocoMOS). The aim was to compare and identify differences in tissue-specific host mucosal immune expression and mucosal microbiota composition and function, while also highlighting dietary influences on the same endpoints and finally measuring the culmination of these effects with an experimental salmon louse (*Lepeophtheirus salmonis*) challenge.

## Results

### Growth performance

Water quality of the natural inflowing water was in acceptable ranges for the duration of the study and temperature ranged from 19˚C at the beginning of the study to 11˚C at the conclusion (Additional file 1: Figure S1). At the conclusion of the 12-week trial, fish weighed 646.5 g ± 35.8 (mean ± SD), with 288.2 g ± 38.3 of growth over the trial. No difference in weight gain was detected by dietary treatment (ANOVA, *p* = 0.4014). Fish grew 311.85 ± 38.16, 278.52 ± 25.34, 280.47 ± 35.26, 282.12 ± 49.95 for the Control, MOS, CoconutOil, and CocoMOS groups, respectively.

### Microbiota analysis

Following post-clustering ASV curation and removal of mitochondria and chloroplast reads, 3,087 unique ASV were identified across the 226 samples in the full dataset (8 diet, 3 water, 72 gill, 71 gut, and 72 skin sample). Samples below the acceptable sequencing depth (> 20,000 reads) were removed (4 gut and 3 gill), with the remaining 209 mucosa-associated samples (69 gill, 68 gut, 72 skin) having 50,1434 ± 23,196 (mean ± SD) ASV assigned reads sample^−1^. After removal of spurious ASV (relative abundance < 1 e^−5^), 2,378 unique ASV were detected among the mucosa-associated sampled. Triplicate positive control microbial community samples showed the workflow to accurately identify bacterial taxonomy at genus or species level, with only a few reads in one positive control being assigned to the genus *Allivibrio* (< 0.001%) which was not included in the positive community but was highly abundant among experimental samples. In addition, positive control data showed good concordance with theoretical relative abundance (Additional file 1: Figure S2A). Negative controls yielded few 16S rRNA gene sequence reads (2,845 ± 1,452; mean ± SD) compared to experimental samples (Additional file 1: Figure S2B) suggesting background contamination did not influence data analysis.

#### Alpha diversity

**Fig. 1:**
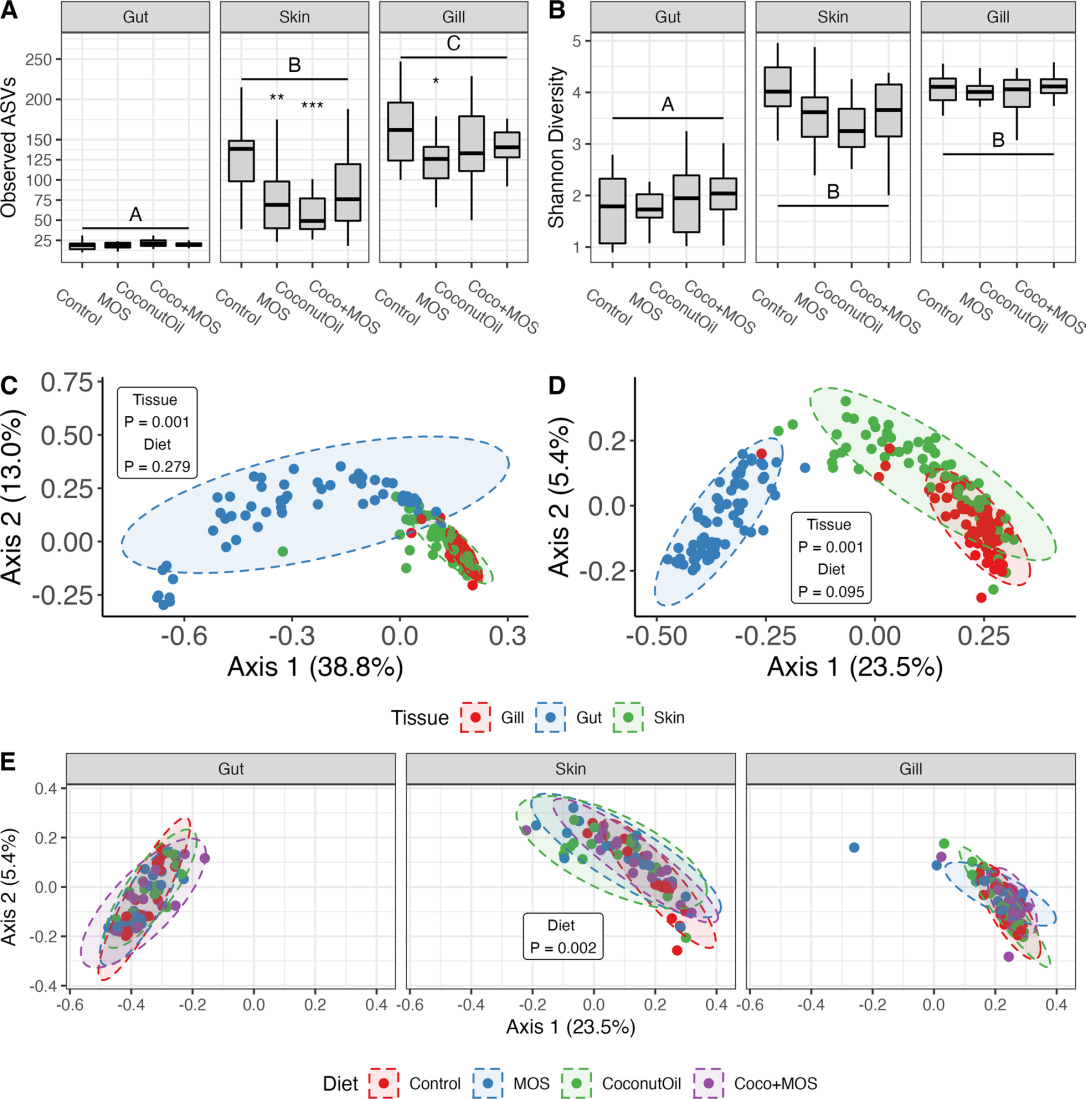
Microbiota composition detected across mucosal tissues of Atlantic salmon fed diets supplemented with functional ingredients. Fish received a control diet (Control), a 1% mannan oligosaccharide supplementation (MOS), a 96% lipid replacement using coconut oil (CoconutOil) or a combination of the two treatments (CocoMOS). Alpha diversity was tested by two-way ANOVA with tissue showing global effects on richness (**A**) and Shannon diversity (**B**). Due to tissue-diet interactions, dietary effects on richness and diversity within each tissue were also tested by one-way ANOVA and Dunnett’s post-hoc (* *p* ≤ 0.05, ** *p* ≤ 0.01, *** *p* ≤ 0.001). Beta diversity is displayed by tissue using principal co-ordinates analysis (PCoA) of weighted (**C**) and unweighted (**D**) UniFrac distances with statistical values taken from PERMANOVA. Within tissue dietary effects on microbiota composition were only detected in the skin according to both weighted (*p* = 0.039) and unweighted UniFrac (**E**). Pairwise PERMANOVA showed the MOS and Coconut oil diets to significantly altering communities relative to the control in unweighted UniFrac (**E**)

Rarefaction analysis indicated all samples were sequenced deeply enough to reach an asymptote in bacterial richness (Additional file 1: Figure S3). In terms of alpha diversity, both observed ASV richness and Shannon diversity, were calculated by individual before removing outliers using Tukey’s method (> 1.5 * IQR). Both richness and diversity were tested by two-way ANOVA using a linear mixed effects model fit to tissue, diet, and tissue-diet interaction, while controlling for random tank effects nested within diet. Results showed both microbiota richness and diversity to be highly different across mucosal tissues (*p* ≤ 0.001), with a pairwise Tukey’s post-hoc showing the gut microbiota to have significantly (*p* < 0.001) less microbial richness and diversity than the exterior microbiota of the gill and skin, while the gill showed even greater richness than the skin (*p* = 0.018). Diet was not found to have a global effect on richness (*p* = 0.428) or diversity (*p* = 0.893), although tissue by diet interaction effects were detected on richness (*p* = 0.002) (Fig. 1A, B). Due to interaction effects, dietary effects on alpha diversity were further evaluated using one-way ANOVA to independently test for dietary effects within tissues, with significant effects detected in the skin (*p* = 0.002) and gill (*p* = 0.048). In the skin, a Dunnett’s post-hoc identified MOS (*p* = 0.005) and CoconutOil (*p* < 0.001) as significantly reducing microbiota richness compared to the control, while in the gill only the MOS diet showed a significant reduction in richness (*p* = 0.016). Interestingly, the dietary treatments were not shown to influence gut richness (*p* = 0.246) or diversity in any tissue (*p* > 0.053).

#### Beta diversity

Comparisons of overall microbial communities among samples were made by first calculating phylogenetically informed weighted (wUniFrac) and unweighted UniFrac (uwUniFrac) sample distances. Multivariate dispersion (homogeneity of variance) was tested individually by tissue and diet, with tissue groups identified as having unequal dispersion in both wUniFrac and uwUniFrac (*p* < 0.001) (Fig. 1C, D). According to abundance weighted distances, the gut microbiota showed notable variance (Fig. 1C), while presences-absence distance showed the greatest variance among the skin samples. Beta dispersion was not influenced by diet (*p* ≥ 0.710). Adonis2 [58] was used to perform a PERMANOVA to test main effects of diet, tissue, and interaction effects, while accounting for nested tank-effects, using 999 permutations. Tissue showed highly significant effects (*p* ≤ 0.001) on both weighted and unweighted UniFrac distances (Fig. 1C, D), while no global dietary or interaction effects were detected. Pairwise-PERMANOVA [31] ran between the three tissues detected highly significant differences among all pairwise tissue comparisons using both distance metrics (Fig. 1C, D; FDR-corrected *p* ≤ 0.001). Tissue specific influences of diet on beta diversity were further tested by running a PERMANOVA separately on each tissue by modeling the effects of diet, while controlling for tank-effects. Only the skin microbiota showed within tissue dietary effects with significant differences detected in both weighted (*p* = 0.039) and unweighted (*p* = 0.002) UniFrac distances. Although, pairwise dietary differences in skin microbiota were only detected using unweighted metrics, as MOS (*p* = 0.042) and CoconutOil (*p* = 0.024) diets showed multivariate centroid deviation compared to control samples.

#### Microbiota composition and differential abundance

Bacteria from 32 different phylum were detected in the study, with the top five most abundant phyla being *Proteobacteria*, *Bacteroidetes*, *Firmicutes*, *Actinobacteria*, and *Verrucomicrobia* for the gut, gill, diet, and water samples (Additional file 1; Figure S4). In the skin samples the same phyla were present, however, *Patescibacteria* replaced *Verrucomicrobia* as the fifth most abundant phyla. At the phylum level, taxonomic composition was similar among all sample types (Additional file 1: Figure S4). In the diet samples, 265 unique ASV were detected, while 637 unique ASV were detected in water samples. A total of 448, 1,818, and 1,604 unique ASV were detected in the gut, skin, and gill mucosa, respectively (Additional file 1: Figure S4). The gill and skin mucosa had the greatest overlap in microbial composition with nearly 600 shared ASV, and these two tissues also shared over 450 ASV with the water microbiota (Additional file 1: Figure S4). Surprisingly, the gut mucosal microbiota shared more ASV with the gill than any other sample type, including diets (Additional file 1: Figure S4).

**Fig. 2:**
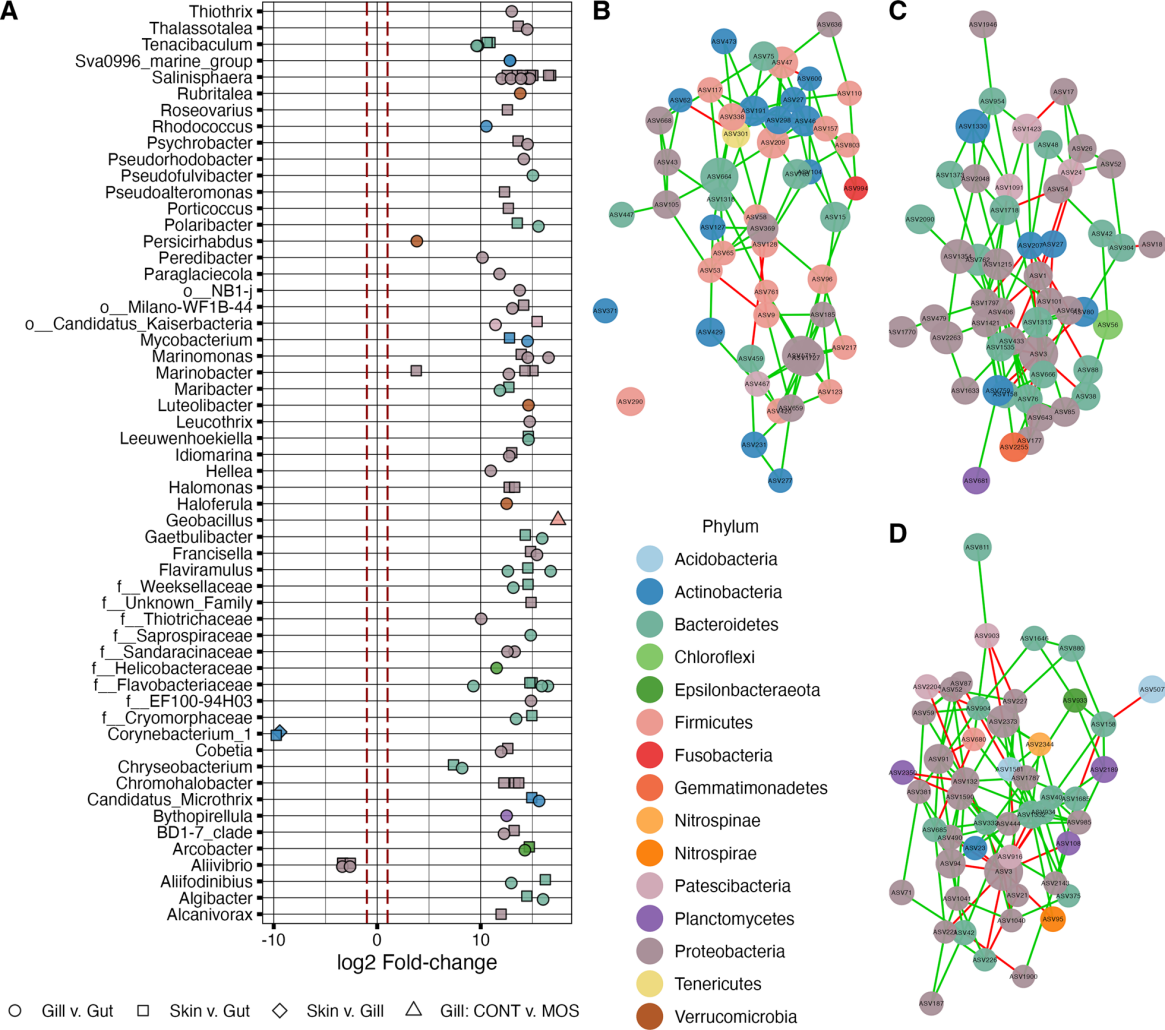
Keystone microbiota of Atlantic salmon associated with mucosal tissues and dietary treatments according to differential abundance testing and network analysis. A log2-fold-change plot (**A**) shows the results of pairwise differential abundance (DA) conducted between tissues, while controlling for diet using DESeq2 (FDR corrected q ≤ 0.05 and log2-fold change|≥ 1). Within tissue dietary effects were also tested, with only one dietary DA ASV identified (Gill: Control v. MOS) (**A**). Bacterial genera are listed on the y-axis, points are colored by phylum, and shape identifies the pairwise treatment comparison for which the ASV showed DA. Positive fold-changes indicate an increased abundance in the first group in the comparison, and vice versa. Microbiota networks (**B**–**D**) depict the top 50 most connected ASV (nodes) according to sparse inverse co-variance networks reconstructed from the gut (**B**), gill (**C**), and skin (**D**) microbiota datasets. Network nodes are colored by phylum, while node size is positively correlated with Laplacian centrality, and edges are colored by positive (green) and negative (red) covariance relationships

Differential abundance (DA) testing at the ASV level was first used to determine whether the abundance of bacteria could discriminate between mucosal tissue (Fig. 2A). Fifty-nine ASV were identified as DA (FDR-corrected *p* ≤ 0.05; |log2 fold-change|≥ 1.0) between the gut and gill, 50 ASV between the gut and skin, and only one between the skin and gill microbiota. Of the DA ASV identified between the skin vs. gut and gill vs. gut, 34 of those were common, and the one ASV identified as DA between skin vs. gill was not differentially abundant among other tissues comparisons (Additional file 1: Figure S5). Dietary influences on microbiota abundance were then tested within each tissue as well, with the gill mucosal microbiota showing the only significant within tissue dietary differential abundance. In the gill, the MOS diet was found to increase the abundance of a single ASV in the genus *Geobacillus* (Fig. 2A) in comparison to the control diet.

#### Network analysis

Because DA analysis identified nearly no impact of diet on microbial abundance within tissues, network reconstruction was conducted at the tissue level by combining data from all dietary treatments to detect tissue-specific patterns of microbial co-association and site-specific keystone bacteria (Fig. 2B–D). Centrality analysis was conducted to quantify connectivity of nodes within networks and multiple network centrality measures were tested on each network, with the most informative metric determined using the package CINNA [3]. Laplacian centrality, which showed high correlation with degree centrality (cor ≥ 0.962), was identified as the most effective centrality measure, and was used to plot, analyze, and compare microbial networks (Fig. 2B–D). The gut microbiota network (Fig. 2B) indicated 614 covariance associations between ASV, with 600 positive associations (97.7%). The gill (Fig. 2C) and skin (Fig. 2D) networks showed much higher degrees of connectivity, with 9,973 and 7,904 network edges, of which 8,646 (86.7%) and 6,941 (87.8%) were positive, respectively. The top 50 keystone species were identified from the tissue specific networks by ranking ASV (nodes) by Laplacian centrality (Additional file 2: Table S1-S3). In order, the top five ASV in terms of centrality in the gut were assigned to *Shingomonas* (ASV1717), *Shingobacterium* (ASV664), and *Pseudomonas fluorescens* (ASV1727), *Facklamia* (ASV209) and *Facklamia tabacinasalis* (ASV 47) (Fig. 2B); *Escherichia/Shigella* (ASV3), *Hyphomonas* (ASV1354), *Brevibacterium* (ASV1330), f_Rhodobacteraceae (ASV2263) and an unclassified c_Bacteroidia (ASV762) in the gill (Fig. 2C); and *Escherichia/Shigella* (ASV3), *Shewanella* (ASV2373), o_Gammaproteobacteria_Incertae_Sedis (ASV94), *Chromohalobacter* (ASV91) and *Halomonas* (ASV132) in the skin (Fig. 2D).

#### Functional predictions

**Fig. 3:**
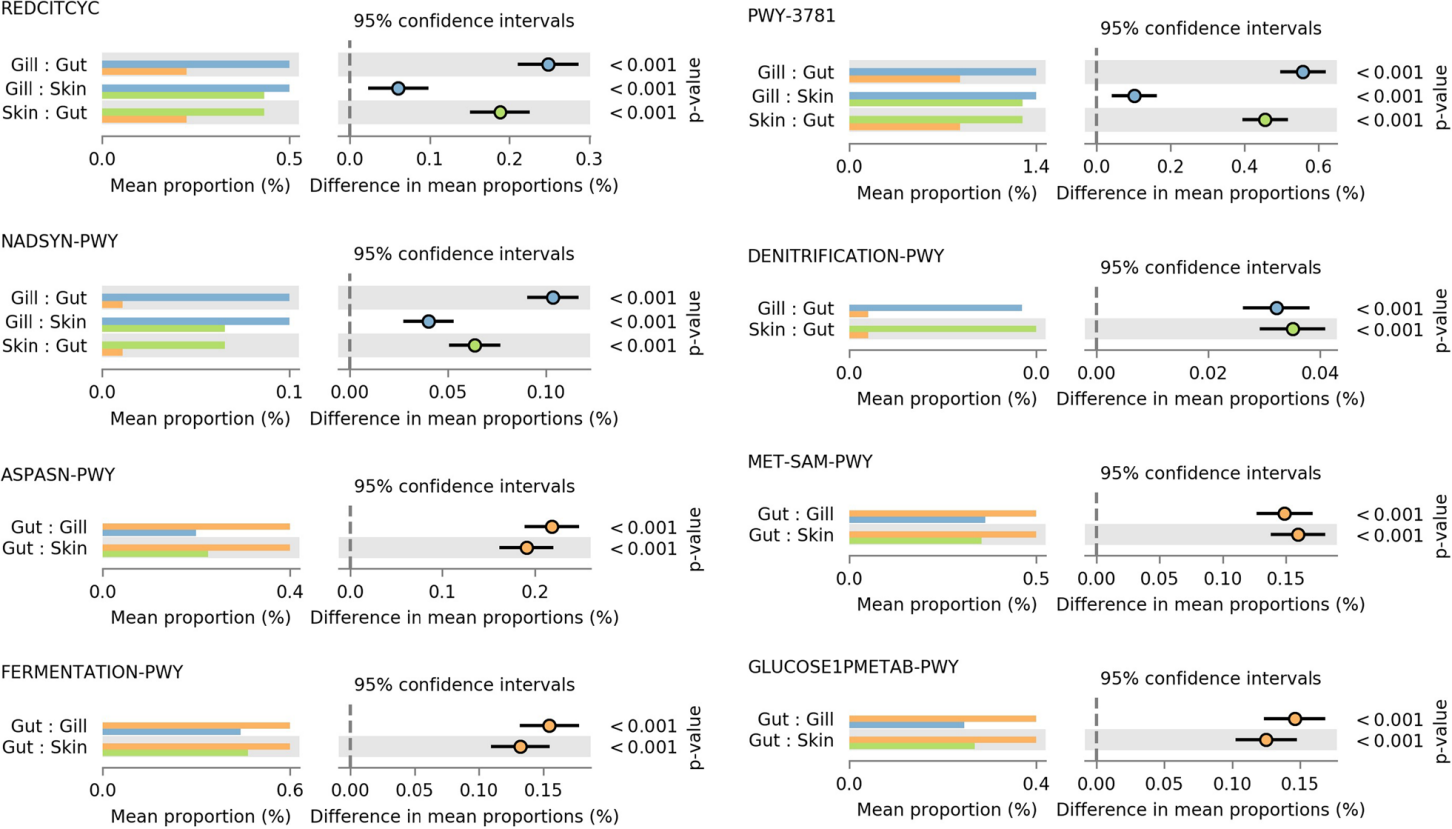
Pairwise tissue-specific differences in inferred metagenomic pathways for Atlantic salmon mucosal microbiota. Metagenomic functions were inferred using PICRUSt2. Pathway abundance was compared across tissues using a Kruskal Wallis test followed by a Tukey’s post-hoc with BH-FDR corrections. Significant differences in pathways abundance were considered at *p* < 0.01 and effect size > 0.5. Out of 404 inferred MetaCyc pathways, 60 showed significant differences across tissue, with only a subset of those shown here. A full list of all differentially abundant inferred metagenomic functions and pathways can be found in Additional file 2: Supplemental Table S4-S6. REDCITCYC—reductive TCA cycle; NADSYN—NAD synthesis; ASPASN-PWY—superpathway of L-aspartate and L- asparagine; FERMENTATION-PWY—mixed acid fermentation; PWY3781—aerobic respiration I; DENITRIFICATION-PWY—nitrate reduction I; MET-SAM-PWY—superpathway of S-adenosyl-L-methionine biosynthesis; GLUCOSE1METAB-PWY—glucose and glucose-1-phosphate degradation

When metagenomic functions from the ASV detected in the three mucosal tissues were inferred using PICRUSt2 [20], a total of 6,884 KEGG orthologs (KO) were detected, with 1,104 KO showing significant differences by tissue (FDR-corrected *p* < 0.01; Effect-size > 0.5) (Additional file 2: Table S4). Inferred KEGG Enzyme Commission (EC) functions showed 362 differential metagenomic functions across tissues out of the 2,179 total inferred EC codes (Additional file 2: Table S5). Statistical testing of functional MetaCyc pathways identified 60 out of 404 inferred pathways to be significantly different across the gut, skin, and gill microbiota (Fig. 3; Additional file 2: Table S6).

### Host gene expression

**Fig. 4:**
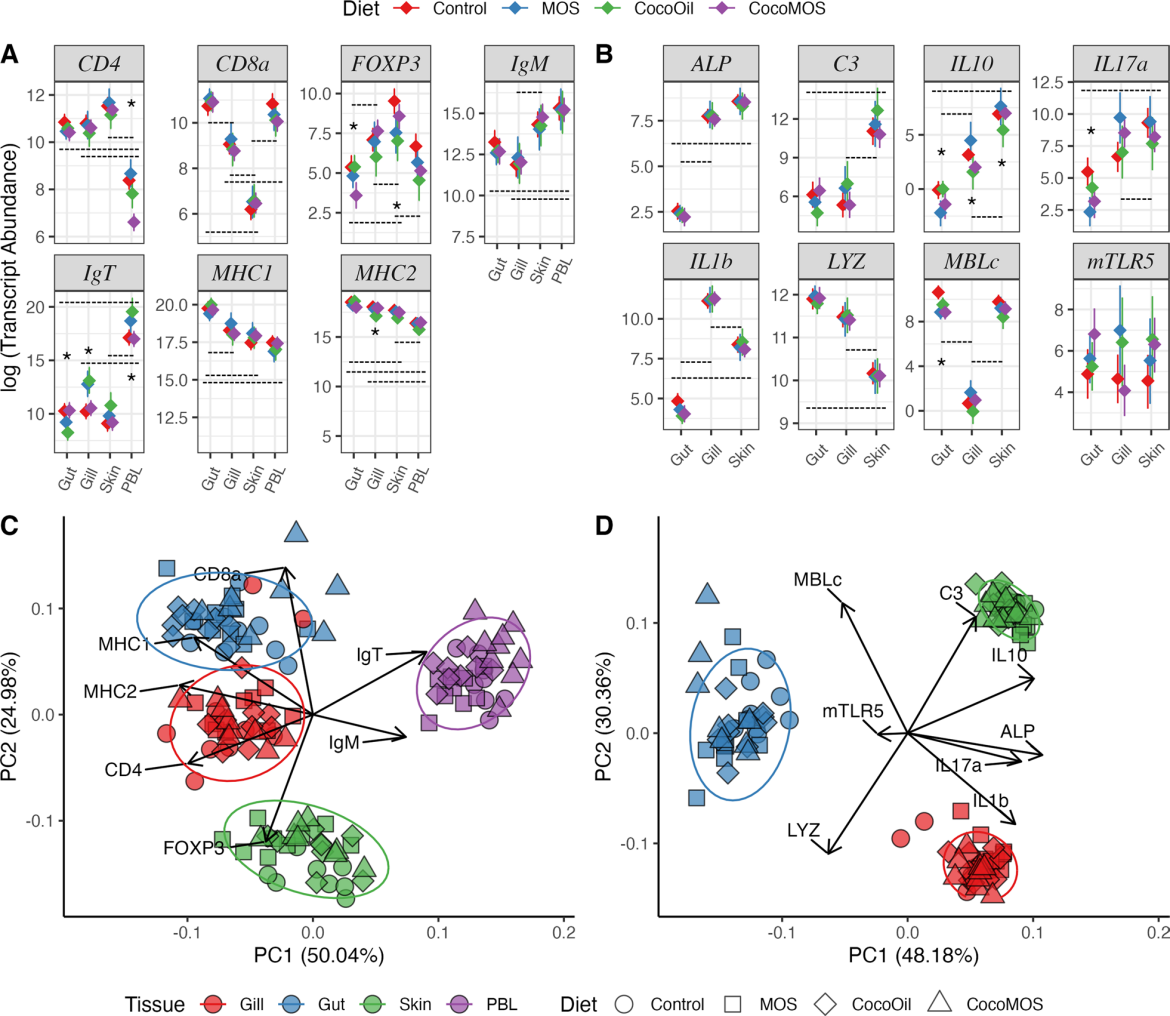
Immune gene expression across mucosal tissues of Atlantic salmon fed diets supplemented with functional ingredients. A set of systemic-adaptive-immunity genes (**A**, **C**) were assayed in the gut, gill, skin and peripheral blood lymphocytes (PBL), while a set of mucosal-innate immunity markers (**B**, **D**) were assayed in the three mucosal tissues. Transcript abundances, shown on a log scale, were inferred from a global Bayesian model using efficiency corrected qPCR data (**A**, **B**). Dashed lines show a significant pairwise difference between tissues among fish receiving the control diet (FDR corrected *p* ≤ 0.05), while significant pairwise differences in expression between two or more dietary treatments within a specific tissue is denoted by *. Principal components analysis plots (**C**, **D**) show the multivariate sample ordinations based on the systemic-adaptive-immunity genes (**C**) and mucosal-innate-immunity genes, with overlaid eigenvector loadings indicating the contribution of each gene

Half of the tanks included in the microbiota analysis were also processed for host gene expression analysis (3 tanks treatment^−1^), with RT-qPCR analysis conducted on all three tissues (gill, gut, and skin). Peripheral blood leukocytes (PBL) were also obtained for gene expression analysis following hypotonic lysis [33] of whole blood samples collected from each fish at the time of sampling to evaluate dietary effects on systemic-adaptive immune genes. Gene expression was analyzed under a Bayesian framework using soft-normalization priors based on reference genes in the MCMCqPCR package [51], and outlier samples were detected and removed based on global models for a set of systemic-adaptive or mucosal-innate genes. The final systemic-adaptive gene set included 140 samples (36 gill, 35 gut, 34 skin, 35 PBL samples), while mucosal-innate gene set included 105 samples (36 gill, 35 gut, and 34 skin samples). Tissue-specific differences in gene expression were detected for all genes assayed apart from membrane Toll-like receptor 5 (mTLR5), which showed high levels of intra-tissue variability (Fig. 4A, B). Within a tissue, all pairwise dietary treatment comparisons were also tested for significance with BH-FDR correction. Within the PBL, the CocoMOS diet group had significantly reduced expression of CD4 compared to all other diets. Dietary effects on expression of FOXP3 were detected in the gut (Control vs. CocoMOS and CoconutOil vs. CocoMOS) and skin (Control vs. CoconutOil). Expression of the mucosal immunoglobulin (IgT) showed dietary effects in the gut (Control vs. CoconutOil and CocoMOS vs. CoconutOil), gill (Control vs. MOS, Control vs. CoconutOil, CoconutOil vs. CocoMOS, and MOS vs. CocoMOS), and PBL (Control vs. CoconutOil and CoconutOil vs. CocoMOS). Gill expression of MHC2 was influenced by diet (Control vs. CoconutOil). Expression of IL10 at the gut (Control vs. MOS and MOS vs. CoconutOil), gill (Control vs. CocoMOS, MOS vs. CoconutOil, and. MOS vs. CocoMOS), and skin (MOS vs. CoconutOil) showed dietary effects, however, IL10 expression in the gut was near the lower limit of detection for the assay and may be less reliable. Dietary effects of IL17A expression were detected in the gut with a significant difference between Control and MOS diets. In the gut, expression of mannose binding lectin type-C (MBLc) was significantly reduced by each diet in comparison to the control diet.

#### Multivariate analyses of gene expression profiles

Principle components analysis (PCA) using single-value decomposition was conducted to visualize the multivariate relationship between samples from various tissues and dietary treatments. The PCA showed good separation by tissue with some clustering by dietary treatment within tissues as well for both the systemic-adaptive and mucosal-innate immune gene sets, with loading vectors indicating genes involved in multivariate separation (Fig. 4C, D). Normalized qPCR data were then used to calculate sample-wise Manhattan distance matrices for multivariate analyses of gene expression profiles of systemic-adaptive and mucosal-innate immunity gene sets. Dispersion by tissue (*p* = 0.117) and diet (*p* = 0.826) was homogeneous for the systemic-adaptive immunity gene set, although multivariate dispersion was found to vary by tissue (*p* = 0.001), but not diet (*p* = 0.459) in the mucosal-/innate-immunity gene set. Multivariate analysis of both systemic-adaptive and mucosal-innate gene expression profiles showed highly significant effects of tissue (*p* = 0.001), diet (*p* ≤ 0.032), and tissue-diet interaction (*p* = 0.001) according to PERMANOVA. Pairwise-PERMANOVA indicated a highly significant difference in expression profiles (*p* ≤ 0.001) between each tissue according to both gene sets, though no pairwise differences in multivariate expression profiles were detected according to dietary treatments (*p* ≥ 0.57).

### Host–microbiota interaction

**Fig. 5:**
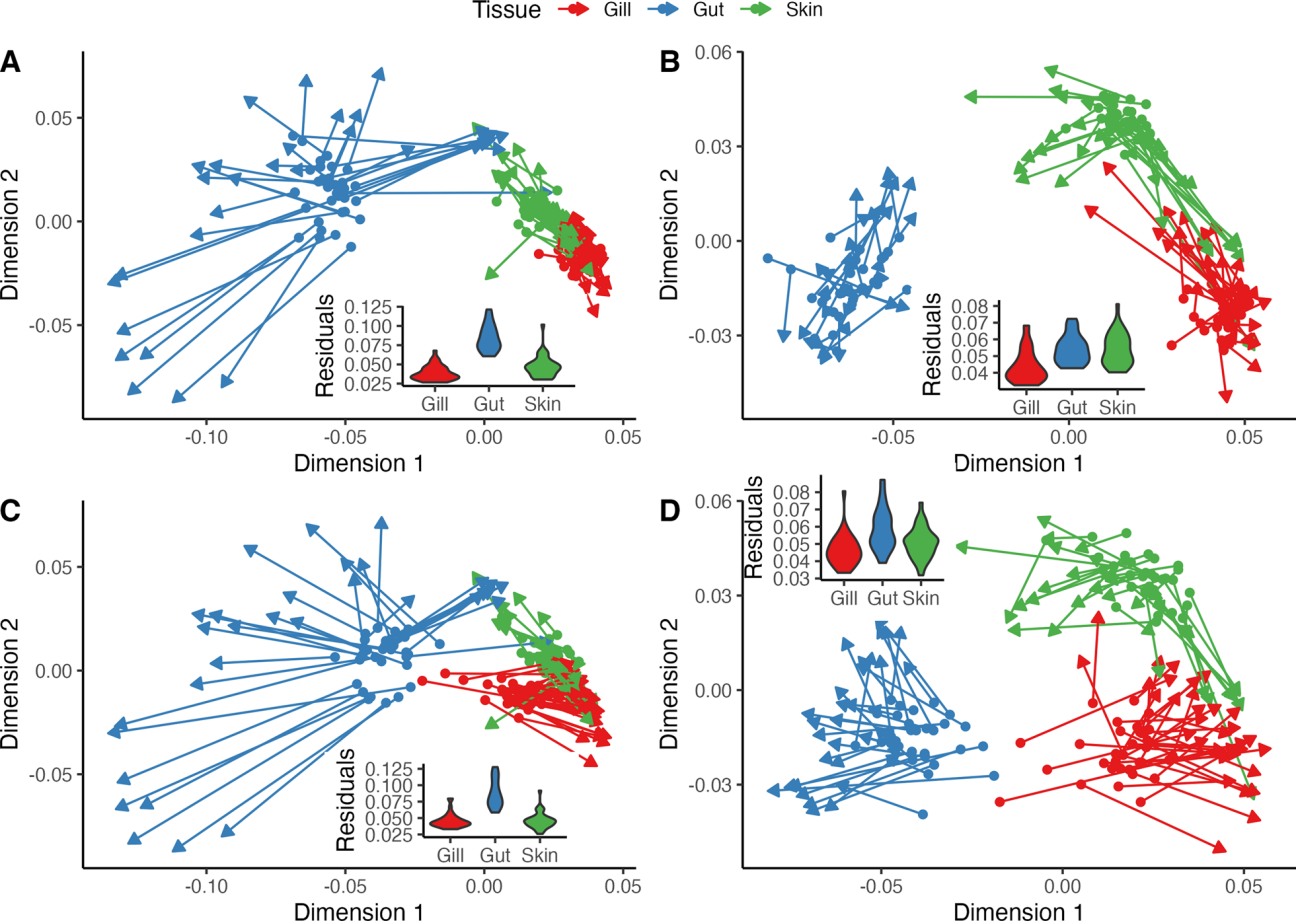
Multivariate Procrustes analysis comparing microbiota composition to host immune gene expression profiles across mucosal tissues of Atlantic salmon. Principal co-ordinates analysis (PCoA) sample ordinations of abundance-weighted (**A**, **C**) and unweighted (**B**, **D**) microbiota phylogenetic composition (UniFrac) (arrows) were mapped to sample ordinations based on host mucosal-innate (**A**, **B**) or systemic-adaptive immune (**C**, **D**) gene expression profiles (points) taken from the same set of samples. Longer lines between a sample gene expression eigenvalue (points) and its concurrent microbiota eigenvalue (arrows) indicates greater discordance between datasets for that sample. Inset violin plots display the distribution of Procrustes residuals by tissue for each plot. Significant correlations (*p* < 0.001, 999 permutations) were detected in all comparisons

To assess associations between host gene expression and microbiota communities, symmetrical Procrustes analysis was used to compare multivariate sample ordinations based on tissue-specific host immune gene expression profiles (Manhattan distances) to sample-matched ordinations of microbiota phylogenetic composition (UniFrac distances) (Fig. 5). Because of the reduced sample size in the gene expression dataset and the independent removal of outliers in each dataset, the qPCR and microbiota data were trimmed to include only gut, gill, and skin samples present in both datasets (35 gill, 33 gut, 34 skin samples). Procrustes analysis showed multivariate sample-sample variance in microbiota composition to be highly correlated (*p* < 0.001) with sample ordinations of host gene expression profiles using both the mucosal-innate and systemic-adaptive (Fig. 5C, D) gene sets. The mucosal-innate gene set showed slightly higher levels of correlation with both abundance-weighted (m^2^ = 0.383, cor = 0.785, *p* = 0.001) and -unweighted (m^2^ = 0.271, cor = 0.854, *p* = 0.001) microbiota composition (Fig. 5A, B). Although, the systemic-adaptive dataset showed a similar level of multivariate congruency with wUniFrac (m^2^ = 0.424, cor = 0.759, *p* = 0.001) and uwUniFrac (m^2^ = 0.286, cor = 0.845, *p* = 0.001) microbiota ordinations (Fig. 5C, D). Residuals from Procrustes analyses indicated that, compared to the gill and skin, gut samples consistently had the greatest disconcordance between host immune expression and microbiota ordinations (Fig. 5).

### Sea lice challenge

A sea lice challenge with infective *L. salmonis* copepodids, following the diet trial, yielded mild levels of infection (18.9 ± 9.8 lice fish^−1^; mean ± SD) in line with sea lice counts from previous experimental challenges [43]. Functional feeds showed no effect on sea lice resistance in triplicate common garden challenges. When corrected for diet-trial tank and challenge tank effects, right-side sea lice density (0.27 ± 0.17,mean ± SD) and lice surface-area^−1^ (0.028 ± 0.017) showed no significant difference by dietary treatment (ANOVA; *p* = 0.614 and *p* = 0.610, respectively).

## Discussion

The supplementation of functional feed ingredients in aquaculture is often touted as a means to improve fish growth performance and overall health, however, previous studies on the use of functional feeds in fish often yield inconsistent finding. In our study, 1% dietary MOS supplementation did not affect fish growth or sea lice resistance. A similar study also showed 1–2% MOS supplementation in adult Atlantic salmon to have no effect on salmon louse susceptibility, although, growth, feed efficiency, and resistance to soybean-meal induced intestinal enteritis were improved [67]. Conversely, a recent trial (44 days) evaluating 0.4% MOS supplementation in Atlantic salmon found no significant impact on growth performance [43], yet salmon louse counts were reduced. Contradictory results on phenotypic responses to MOS (i.e., growth and sea lice resistance) are likely explained by differences in fish species, life-history, and age, ingredient dose, form, and duration, as well as husbandry practices and environmental conditions, all of which confound comparisons across studies [76]. Additionally, the coconut oil diet in our study, alone or in concert with MOS supplementation, did not affect fish growth or sea lice resistance either. One of the only other in vivo studies evaluating high levels of dietary coconut oil inclusion in salmonid diets showed coconut oil to also have no effect on growth rates or feed efficiency in rainbow trout (*Oncorhynchus mykiss*) [48]. While dietary effects on fish growth were not significant, all diets supplemented with functional ingredients yielded numerically lower growth than the control diet. This may be partially explained by energetic costs associated with immunomodulatory effects, as our evaluation of central and peripheral immune expression and mucosal microbiota of the gut, gill, and skin revealed multiple dietary effects at the molecular level.

### Dietary effects of functional ingredients on mucosal microbiota

In this study, an array of microbial-ecology metrics related to the gill, gut, and skin microbiota were tested for dietary effects to better understand impacts of functional feeds on the physiology of mucosal tissues of Atlantic salmon. We showed the MOS diet to reduce bacterial richness in the skin and gill microbiome of Atlantic salmon (Fig. 1A). In rainbow trout, Dimitroglou et al. [19] showed MOS to reduce gut microbial species richness, yet in gilthead sea bream (*Sparus aurata*) the ingredient increased bacterial richness and diversity when combined with fishmeal-based diets and had no effect when combined with soybean-meal based diet [18]. In our study, the MOS diet also shifted the overall bacterial communities of the skin compared to the control (Fig. 1E). A similar diet related shift in skin microbiota was detected in wild coral reef fish, which was proposed to be due to dietary impacts on skin mucus and metabolite production [13], effects which have also been documented in Atlantic salmon receiving MOS supplementation [43, 53]. Furthermore, in our study the MOS diet was responsible for the only dietary impact on tissue-specific microbiota abundance, significantly increasing the abundance of a single ASV in the genus *Geobacillus* (Fig. 2). *Geobacillus* was recently detected as differentially abundant in the gut microbiome of rainbow trout, where it was significantly reduced by a soybean-meal based diet compared to a traditional fishmeal diet [7]; however, this genus of bacteria is typically thermophilic and may therefore be transient non-functional bacteria within the cold-water rearing environment of salmonids.

Like the effects seen from the MOS diet, the coconut oil diet in our study also reduced the bacterial richness and shifted the overall microbial communities of the skin relative to control groups (Fig. 1). To date, no comparable study has evaluated in-vivo impacts of high inclusion of coconut oil on microbiota, yet the in-vitro antimicrobial effects of fatty acids found at high concentrations in this lipid source (i.e., lauric and caprylic acid) could partially explain the reduction in skin microbiota richness. Although, following this mechanism of action, one would expect to see effects on the gut microbiota as well, which were absent in this study. In fact, the lack of dietary impacts overall on the gut microbiota was somewhat surprising in this study. Most studies evaluating functional feed effects on the microbiome are relegated to the gut and are conducted in juvenile fish fed the ingredient over short periods, while our study involved sub-adult fish (358.3 ± 17.8 g initial weight) sampled after a twelve-week feeding trial. The homeostatic microbiome of larger, older fish is likely more stable [8] and therefore less susceptible to the modulation by functional ingredients that have been observed in juvenile fish [18, 29]. Moreover, Gajardo et al. [26] showed the digesta-associated microbiota of Atlantic salmon to be significantly more diverse and susceptible to dietary alterations than the mucosa-associated gut microbiota. This may explain the lower richness detected in the gut in our study, as well as the lack of dietary effects in our mucosa-associated gut microbiota samples. The dietary combination of functional ingredients in this study (CocoMOS) showed no significant effects on any microbiota metrics across all tissues assayed, despite the two ingredients affecting skin richness and composition when fed independently. This suggests interactions between these two ingredients is not additive, and certainly more microbiota research on interaction effects of functional dietary ingredients is needed.

### Dietary effects of functional ingredients on immune expression

We compared expression of seven systemic-adaptive immune genes (Fig. 4A) and eight mucosal-innate genes (Fig. 4B) to evaluate the influence of functional feeds on Atlantic salmon immune regulation and response. Here, the MOS diet was shown to primarily alter expression of genes involved in innate immune pathways, with the MOS diet only significantly altering one adaptive immune gene (IgT) in the gill. The MOS diet reduced expression of inflammation regulating cytokines (IL10 and IL17a), as well as mannose-binding lectin protein c (MBLc) in the intestine. A similar dose (0.6%) of MOS fed to European sea bass (*Dicentrarchus labrax*) was also shown to alter expression of cytokines involved in regulation of intestinal inflammation (IL10, TNFα, and COX-2) [78]. Furthermore, it was somewhat surprising that mannose binding lectin protein c (MBLc) expression in our study was reduced in the gut by all dietary treatments. As a binding lectin produced by epithelial cells, MBLc functions as an opsonin in the lectin-complement pathways and is known to interact with mannose on the surface of various microbes. The addition of a 1% yeast-derived mannan supplement to the MOS and CocoMOS diets was expected to increase MBLc expression in the gut due to an increase in target antigen, although the opposite pattern was observed (Fig. 4B). In a mouse model, a one-week of daily gavage with alpha-mannoside saturated and down-regulated intestinal MBL, leading to a reduced ability to respond to a fungal pathogen challenge [14], however the MOS in that study was administered at much higher concentration and short periods than the present study. This may suggest the MOS treatment diets in this study attenuated intestinal MBLc expression after chronic activation of the lectin complement pathway, although this alone would not explain the reduced expression of intestinal MBLc caused by the CoconutOil diet as well (Fig. 4B).

Unlike the gene expression effects of the MOS diet, the coconut oil replacement diet appeared to mainly alter adaptive immune mechanisms and induced more changes in gene expression than the other dietary treatments (Fig. 4). The coconut oil diet reduced expression of a T_Reg_ marker (FOXP3) in the skin, altered expression of the B-cell mucosal antibody (IgT) in the gut, gill and PBL, and reduced expression a of biomarker for professional antigen presenting cells (MHC2) in the gill (Fig. 4A). The only effect of the coconut oil diet on innate immune expression was a reduction in MBLc expression in the gut, which was true for all functional dietary treatments (Fig. 4B). Despite the lack of a comparable study in fish, a recent study supplementing broiler chicken diets with various lipid sources showed coconut oil to alter serum IgG and IgM concentrations relative to fish oil [4]. Taken with the results of the present study, this may suggest coconut oil has immunomodulatory effects on adaptive-humoral immune responses, though more evidence is needed to confirm these effects.

### Tissue-specific signatures in microbiota

While analyzing microbiota metrics across gill, gut, and skin tissue, clear signatures of each mucosal tissue were identified and differences between tissues greatly outweighed the within-tissue dietary effects observed on the microbiota in the present study. A recent study characterizing the skin, gill, and digesta microbiota of Atlantic salmon reared under different hatchery conditions (recirculating vs. flow-through) also showed the three microbiomes to be significantly different in composition, irrelevant of rearing environment [55]. Additionally, that study found that when reared in a flow-through system, as was done in our study, the microbial richness of Atlantic salmon was highest in the gill, followed by the digesta, and finally the skin of Atlantic salmon. In our study, the number of unique ASV, as well as average bacterial richness was significantly different between all mucosal tissues, with the highest richness detected in the gill, followed by the skin, and the gut (Fig. 1A; Additional file 1: Figure S4E). In addition, Shannon diversity was reduced in the gut, compared to the skin and gill (Fig. 1B). The lack of a significant difference between skin and gill diversity, despite a significant difference in richness, suggests evenness was reduced among the gill microbiota compared to the skin, as Shannon diversity index is a quantitative measure of ASV richness, that also considers the evenness of ASV abundances.

Also in agreement with Minich and colleagues’ previous report [55], our results indicate the bacterial community structure of the gut, gill, and skin mucosa is significantly different from one another according to quantitative (Fig. 1C) and presence-absence UniFrac distances (Fig. 1D). Although, we also showed the gill and skin communities to be much more similar to one another, compared to the mucosal gut microbiota, in terms of alpha diversity (Fig. 1A, B), beta diversity (Fig. 1C, D), differential abundance (Fig. 2A), network structure (Fig. 2C, D), as well as inferred metagenomic function (Fig. 3). Legrand et al. [45] similarly found appreciable overlap between the gill and skin microbiota, despite also detecting tissue-specific signatures, in yellowtail kingfish (*Seriola lalandi*), where 85% of the detected microbiota were shared between those two tissues. Here, an ASV in the genus *Corynebacterium* was the only bacteria identified as significantly more abundant in the gill than in the other two tissues (Fig. 2A). The abundance of this genus was recently identified as the most predicative gut microbiota metric for detecting seasonal dietary shifts in wild three-spine stickleback (*Gasterosteus aculeatus*) [24], although, *Corynebacterium* is also found in the skin and gill microbiome of yellowtail and where its abundance was positively correlated with health [45].

According to tissue-specific microbiota networks, an ASV (ASV3) in the genus *Escherichia/Shigella* had the highest degree of connectivity within the gill and skin microbiota and was among the fifty most highly connected ASV in the gut network as well (Fig. 3; Additional file 2: Table S1-3). Microbes from the genera *Shigella* and *Escherichia* are difficult to discriminate by 16S rRNA gene amplicon phylotyping, hints the combined annotation, though both are enteric pathobionts commonly detected in animal microbiomes. While showing a high degree of network connectivity, this *Escherichia/Shigella* node had predominantly negative covariance interactions with other ASV, which has been observed in networks constructed from the human gut microbiome as well [86]. In addition, it should be noted that two keystone genera highlighted by differentially abundance analysis and microbiota networks (i.e., *Aliivibrio* and *Escherichia/Shigella*) were also detected in our negative control samples (Additional file 1: Figure S2B). However, highly abundant contaminates in on-plate negative-controls often originated from well-to-well contamination from abundant templates in adjacent biological samples [54] and *Escherichia/Shigella* and *Aliivibrio* are both known to be core microbiota of Atlantic salmon [17], suggesting the findings related to those genera are accurate.

### Tissue-specific signatures in immune expression

Differential gene expression analysis of a set of adaptive immune cell biomarkers was used to identify tissue-specific signatures in immune regulation by comparing tissue expression profiles within the control-diet groups (Fig. 4). According to expression levels, CD4 + T_H_ cells were more abundant or transcriptionally active in all mucosal tissues compared to that of the circulating PBL, while CD8 + transcripts derived from T_c_ cells were more abundant in the gut and PBL compared to the skin and gill (Fig. 4A). The PBL showed the highest levels of expression for immunoglobulins (IgT and IgM), suggestive of transcriptionally active circulating plasma B-cells. As an analog to the mammalian mucosal antibody IgA, IgT is responsible for adaptive humoral regulation of pathogens and commensal microbiota on mucosal surfaces of fish, while IgM immune responses are typically highest in circulating sera [82]. As such, the relatively high expression of IgT in the PBL was surprising (Fig. 4A). Although Hu et al. [33] also identified high levels of IgT expression in rainbow trout PBL while optimizing the PBL isolation technique used here, which is likely explained by this sample type being enriched with peripheral lymphocytes. The expression of the major histocompatibility complexes (MHC1 and MHC2) in this study also showed tissue specific differences, with the highest expression of both MHC biomarkers, in order, being the gut, gill, skin, then PBL (Fig. 4). Furthermore, the expression of these important antigen presenting peptides appears to be tightly regulated, as the within-treatment variance observed for these gene was minimal compared to the other genes assayed.

Comparison of innate immunity biomarkers across the three mucosal tissues, identified further tissue-specific signatures. Alkaline phosphatase, a class of isoenzymes involved in a range of cellular, digestive, and immunological roles, is typically found in highest concentrations in the blood originating from hepatic cells, though, ALP is also found in mucosal tissue where it serves a role in inflammation, innate defense, and wound healing [22]. In our study, ALP expression was significantly reduced in the gut compared to the gill and skin, although intestinal ALP expression has been shown to be more focused to the proximal intestine [41], potentially explaining the low level of expression detected here in the distal intestine. Additionally, Johnston et al. [36] isolated ALP isoenzymes from various tissues of Atlantic salmon and showed intestinal ALP to have a significantly different electrophoretic mobility than that of the gonads, bone, kidney, and liver. Therefore, it is possible the intestinal ALP isoenzyme is expressed as a transcript not targeted by the ALP primers used in our present study (Additional file 2: Table S7), highlighting the need for further delineation of isoenzymes and paralogous genes in Atlantic salmon [41]. Complement factor 3 (C3) showed significantly higher expression in the skin compared to the gut and gill in our study (Fig. 4B, D), which slightly diverges from results of Løvoll et al. [47], who showed C3 expression in adult Atlantic salmon to be highest in the liver, followed by the heart, gonads, muscle, intestine, skin, kidney, pylorus, spleen, and finally gill. Furthermore, we showed MBLc expression to be significantly reduced in the gill compared to the gut and skin in Atlantic salmon. Similarly, previous work in channel catfish (*Ictalarus punctatus*) showed the liver to be the tissue with by far the highest levels of MBLc expression, as well as low levels of expression in the intestine, and no detectable expression in the gills [61].

In terms of the cytokine signaling, interleukin 10 (IL10), IL17a, and IL1β expression levels were compared to assess regulation of inflammation across the mucosal tissues (Fig. 4B, D). Expression of the immunosuppressive and often anti-inflammatory cytokine IL10 was significantly different among all the tissues in our study, with highest expression being in the skin followed by gill, while gut IL10 expression was at the limit of detection (Fig. 4B). This finding is in line with Marjara et al. (2012), who also found IL10 expression in the gut to be at or below the limit of detection in Atlantic salmon. Conversely, expression of IL17a, a proinflammatory marker produced by T_H_17 cells with putative roles in the clearance of pathogens and autoimmune responses, was also highest in the skin in our study. Furthermore, proinflammatory IL1β was significantly different among all tissues, with gill showing the highest expression and the gut the lowest. The proinflammatory cytokines, IL1β and IL17A, at least in mammals, are thought to be released following pathogen detection, while more anti-inflammatory responses, such as those resulting from TGFβ, IL8, or IL10, are thought to be activated following interactions with commensal microbes [28]. Viewing our data through this paradigm may suggest that the higher levels of IL10 expression in the skin and gill provides greater immunological tolerance of the more dynamic external mucosal microbiomes as compared to the more intimately regulated intralumenal environment of the gut. These differences in immune regulation across mucosal tissues may then at least partially explain the higher microbiota richness and Shannon diversity, as well as susceptibility to diet induced shifts in ASV abundance or overall microbiota composition, which were observed in the gill and skin samples in our study compared to the gut (Figs. 1, 2). These findings give insights into host regulation of, and response to, mucosal microbiota, though further research is needed to gain a more wholistic understanding of how these interactions influence mucosal physiology in fish.

### Host–microbiota interaction

A phylogenetic alignment based functional prediction algorithm tied to a database containing over 20,000 full length 16S rRNA gene-to-genome mappings (PICRUSt2) was implemented in this study to infer metagenomic functional potential of the tissue-specific microbiota. Differential abundance testing of inferred metagenomic functions was used to highlight trends in putative metagenomic functionality across the gut, gill, and skin to foster future hypotheses regarding the functional significance of these mucosal microbiomes on the physiology of host tissues. Of the inferred metagenomic KO, EC, and higher-level metabolic pathways, 16.0, 16.6, 14.9%, respectively, were detected as differentially abundant across tissues in our analysis. In many cases functions showed microbial adaptations in tissue-specific sites that would likely provide utility to the host. As an example, gills are the primary site of nitrogen excretion in most fish, and here the gill microbiome was shown to possess a greater abundance of genes involved in the denitrification pathways compared to microbes found in the gut (Fig. 3). In agreement, microbes known for their ability to oxidize ammonia, nitrite, and nitrate have previously been detected in the Atlantic salmon skin, gill, and digesta microbiomes [55]. Although, in both cases, it is possible those denitrifying microbes are not highly adapted to fish mucosa and are detected on mucosal tissues as transient environmental microbes from the aquatic environment. Additionally, the gut microbiota were shown here to have a greater ability to conduct anaerobic mixed acid fermentation, while the exterior mucosal sites (gill and skin) were populated with microbiota with a higher prevalence of genes related to aerobic respiration (Fig. 3). The abundance of the fermentation pathway in the gut would suggest those bacteria are generating short chain fatty acids as fermentation byproducts, which are known to serve as an energy source for host enterocytes, as well as being involved in enteroendocrine signaling that modulates cellular proliferation, inflammation, and metabolism [9]. Furthermore, we identified metabolic pathways related to carbohydrate and amino acid metabolism that were predicted to be more abundant among gut microbes, which could aid in digestion and metabolic transformation of nutrients consumed by the host (Fig. 4D, G). Similarly, a study comparing the predicted metagenomic function of gut microbiota from 20 marine species also showed fish gut microbes to putatively possess significant metabolic pathways related to nutrient metabolism that varied with the trophic level of their host [34]. These inferred functions highlight the potential for tissue-specific signatures in metagenomic functions and the potential metabolic roles microbiota play on host physiology. However, accurate inference of metagenomic function from V3-V4 16S rRNA gene amplicons is limited by an inability to distinguish species or strain level functionality and by inherent biases related to reference databases, which are often dominated with entries related to human-associated microbes and lack good representation of environment-specific functionality of aquatic microbes [20]. Therefore, despite our conservative statistical thresholds for declaring differential abundance of metagenomic function (FDR adjust *p* < 0.01 and effect size > 0.5), it still must be noted that the identification of robust signatures of differential metagenomic function using inferred data can be error-prone [20]. Therefore, the trends in inferred metagenomic function observed in this study require further validation using untargeted metagenomic data. As an example, shotgun metagenomic data recently was used by two separate groups to generate metagenomic-assembled-genomes (MAGs) from salmonid gut microbiota and allowed for genome level characterization of novel salmonid-associated *Mycoplasma* species [12, 66], which have previously been detected, somewhat inexplicably, as dominate microbes in the gut of salmonids using 16S rRNA amplicon data, including our study (Fig. 2B, ASV 301). The MAGs from these two studies revealed these previously ambiguous salmonid-associated *Mycoplasma* species lacked virulence factors but were highly adapted to an intracellular niche within the salmonid gut due to their metabolic dependence on host nutrients, while also providing useful metabolic pathways to their host (i.e., chitin degradation, arginine and riboflavin biosynthesis, ammonia detoxification, etc.) [12, 66]. One group took these methods even further to show that functional feed ingredients (i.e., probiotic—*Pediococcus acidilactici*; symbiotic—*P. acidilactici* + galacto-oligosaccharides) specifically reduced the relative abundance and functional significance of these *Mycoplasma* gut microbiota, which yielded significant alterations to the fecal metabolome [65]. This highlights the utility of such data in advancing our understanding of host microbiota interactions in fish and in order for us to gain the knowledge necessary to control and modulate microbiota in a way that provides optimal outcomes in aquaculture more studies using untargeted metagenomic approaches are needed to complement the growing body of 16 rRNA gene marker microbiota studies related to aquaculture species.

In addition to our putative evidence of functional coadaptation of microbiota in a tissue-specific manner that is beneficial to the host, we also used symmetrical Procrustes analysis to determine whether host-gene expression profiles and microbiota composition were correlated across mucosal tissues. Procrustes analysis is a method similar to a Mantel test that evaluates correlations between two multivariate datasets by scaling and rotating sample ordinations to minimize intra-sample multivariate residuals between the two datasets, which has been used previously in a similar manner to evaluate correlations between host gene expression and microbiota composition in zebra finches (*Taeniopygia guttata*) from different environments [79] or humans with different diseases [63]. Here we mapped multi-tissue multivariate PCoA ordinations of host gene expression profiles (adaptive or innate gene set Manhattan distances) to PCoA ordinations based on microbiota composition of those same samples (weighted or unweighted UniFrac distances) and used Monte Carlo label permutations to statistically test the goodness-of-fit (m^2^) between the two datasets (Fig. 5). Highly significant correlations were detected between all host gene expression and mucosal microbiota datasets in our study, suggesting host gene expression and mucosal microbiota composition covary across mucosal tissues of Atlantic salmon (Fig. 5). Although, similar to results seen by van Veelen et al. [79] immune expression showed slightly higher goodness-of-fit with presence-absence based microbiota metrics (Fig. 5B, D) indicating host immune expression may be more dependent on the occurrence of microbes rather than their abundance. Furthermore, consistent differences in Procrustes residuals by tissue (Fig. 5), as well as the tissue-specific differences discussed above, imply host-microbiota interactions and coadaptation slightly vary across the mucosal tissues. While these findings suggest host gene expression and microbiota are interdependent across mucosal tissues of Atlantic salmon, disentangling top-down effects (i.e., host expression regulating microbiota composition) from bottom-up effects (i.e., microbiota shifts altering host expression) will require additional experiments involving manipulations of host expression and experimentally altered microbiota communities. Together, these findings have applied implications on the supplementation of aquaculture diets with functional feed ingredients, while also advancing our basic understanding of host-microbiota-diet interactions in fish and how those interactions vary by mucosal tissues.

## Methods

### Experimental diets and fish husbandry

A twelve-week feeding trial was performed at the USDA-ARS National Cold Water Marine Aquaculture Center (NCWMAC, Franklin, ME, USA). Four experimental diets were produced at the Bozeman Fish Technology Center (Bozeman, MT, USA) using commercial extrusion technology. A control diet (Control) was formulated to match the NCWMAC post- smolt base diet. A mannan-oligosaccharide diet (MOS) was made consisting of the control diet with wheat-flour replaced by BioMOS (Alltech; Lexington, KY, USA) to achieve a 1% (10 g kg-1) inclusion. A third diet was formulated to replace 96% of the lipids (4% fish oil) in the control diet with coconut oil (CoconutOil) and the fourth diet was a combination of the MOS and Coco treatments (CocoMOS). The trial was conducted in a twenty-four-tank system supplied with flow-through natural seawater. Each dietary treatment was administered to six replicate tanks. Twenty individually tagged Atlantic salmon with an average initial weight of 358.3 g ± 17.8 (mean ± SD) were randomly stocked to each tank.

All fish were allowed to acclimate to the system for one month while receiving the control diet. Photoperiod followed natural cycles (Aug.–Nov.) and seawater pumped from an adjacent bay (Taunton Bay, Franklin, ME, USA) followed ambient temperatures (Additional file 1; Figure S1). Water quality, salinity (31.1 ppt ± 0.7; mean ± SD), and temperature were monitored weekly and maintained within acceptable ranges for the duration of the study. Fish were fed using automatic feeders controlled by a continuous dynamic function to supply 110% of maximum expected daily consumption. Growth was assessed at six- and twelve-weeks using bulk tank weights.

### Sample collection

At the conclusion of the twelve-week feeding trial, samples were collected from three fish tank^−1^ (n = 18 diet^−1^) twelve hours post-prandially. Fish were euthanized with tricaine methanesulfonate following AMVA recommendations [42]. Whole blood was collected by caudal venipuncture using a heparinized syringe and held on ice until further processed. Microbiota communities of the gut, gill, and skin were sampled using Whatman OmniSwabs® (GE Healthcare; Chicago, IL, USA). Skin microbiota was sampled by swabbing the left side of fish along the lateral line, between the operculum and caudal peduncle. Gill samples were collected by swabbing between gill arches. Gut microbiota samples were collected by swabbing the mucosa of the distal intestinal tract following careful excision of the intestinal tract and removal of feces. Dietary microbiota samples were collected by homogenizing each treatment diet using mortar and pestle and water microbiota was collected by filtering 1 L of inflow water through a 0.2 µm Supor filter (Pall Corporation; Port Washington, NY, USA). Diet and water microbiota samples were collected in triplicate. All microbiota samples were flash frozen in liquid nitrogen and stored at − 80 °C until further processed. Host tissue was concurrently sampled from each mucosal site for gene expression analysis. Skin tissue samples consisted of a 2 cm2 section of skin excised from between the dorsal fin and lateral line on the left side of the fish. Gill tissue was sampled from the second gill arch on the left side of the fish and a 2–3 cm section of distal intestine was taken 3 cm anterior of the cloaca to serve as the gut tissue sample. All tissue samples were preserved in RNAlater® (Thermo Fisher Scientific; Waltham, MA, USA) and stored at − 80 °C until processed.

### Isolation of peripheral blood leukocytes (PBL)

Whole blood was processed to isolate circulating peripheral blood lymphocytes and leukocytes (PBL) through hypotonic lysis and removal of erythrocytes following methods first described by Crippen et al. [15] and improved by Hu et al. [33]. Briefly, hypotonic lysis was initiated by 1:10 dilution of blood with prechilled ultrapure water for 20 s before returning the solution to isotonicity with 10X Dulbecco’s Phosphate Buffered Saline (DPBS). Cellular debris of the lysed red blood cells was allowed to settle for 10 min before filtering the supernatant through 70 µm cell strainers. The resulting supernatant was centrifuged at 200 × g for 5 min at 4 °C to pellet the PBL. Cells were gently washed twice with 1 × DPBS, prior to storing in RNAlater®. All reagents used in PBL isolation were pre- sterilized (autoclaved and 0.2 µm filtered) to reduce the risk of contaminants that may stimulate or change PBL expression profiles during sample processing.

### 16S rRNA gene library preparation

Microbiota swabs and positive control microbial community samples (ZymoBIOMICS Microbial Community Standard, Zymo Research, Irvine, CA, USA) were homogenized with 0.7 mm garnet beads using a TissueLyser (Qiagen; Hilden, Germany, EU) prior to isolating DNA using a QIAmp 96 PowerFecal QIAcube HT Kit and QIAcube HT (Qiagen) liquid handler following manufacturer recommended procedures. DNA purity and concentration were assessed on a Nanodrop 2000 (Thermo Fisher Scientific) and due to low purity among some samples, as suggested by 260/230 spectrophotometric ratios, all samples were cleaned and concentrated using a gDNA Clean and Concentrate Kit (Zymo Research). Raw DNA samples were normalized by fluorometry (Quant-iT PicoGreen dsDNA kit; Thermo Fisher Scientific) and used as template for preparation of V3-V4 16S rRNA gene sequencing libraries following a strategy similar to that detailed by Fadrosh et al. [21] using 341F and 785R primers [37]. The 16S rRNA gene amplicons were prepared by duplicate 25µL PCR reactions consisting of 30 cycles of PCR using Phusion HiFi Hot Start II Mastermix (Thermo Fisher Scientific), target-specific primers (500 nM each, Ta = 60 °C) and 10 ng of template DNA. No-template amplification negative controls were included with each plate, with DNA template replaced with molecular grade water.

Duplicate PCR products were pooled by sample and confirmed by electrophoresis on 2% agarose gel prior to purification with DNA purification beads (0,8X; MagBio; Gaithersburg, MD, USA). Resulting PCR products were diluted two-fold prior to adding 1 µL as template for eight cycles of PCR in 50 µL reactions with 200 nM custom barcode-indexes (Ta = 72 °C). Resulting libraries were again confirmed by electrophoresis and purified with magnetics beads (0.8X) prior to equimolar pooling according to fluorometry (Quant-iT PicoGreen dsDNA kit; Thermo Fisher Scientific). All negative control samples produced libraries with a final concentration below 1 ng µL^−1^, yet 5 µL of each NTC library were spiked into the final sequencing library to further assess potential background contamination. The final pool was analyzed on a DNA 1000 chip (Agilent Technologies; Santa Clara, CA, USA) to confirm library size and final quantification was done using a Kapa qPCR Illumina Quantification Kit (Roche; Indianapolis, IN, USA). Sequencing was done in-house at the Hagerman Fish Culture Experiment Station on a full MiSeq (Illumina) 600-cycle v3 sequencing. Sequencing required spiking-in three custom sequencing primers (BAMF-CS1, BMF-CS2, BAMF-CS2rc) which were modified by the University of Idaho Genomics Resources Core to match the thermocycling parameters of an Illumina MiSeq by incorporation of locked-nucleic acids.

### Microbiota analyses

To ensure the highest accuracy and reliability of microbiota richness and diversity metrics, a recommended workflow for amplicon data processing [25] was followed to correct and remove any PCR- and sequencing-biases. First, DADA2 [10] was employed for error-correction and processing of raw reads into bacterial amplicon sequence variants (ASV). Reads were truncated (Forward—275 bp, Reverse—215 bp, quality filtered (2 expected errors, error-corrected, and merged (min overlap = 20 bp; merged length ≥ 400 bp. Chimeric sequences were removed, and taxonomy was applied against the Silva nr_v132 rRNA database [64]. ASV were then further clustered by sequence similarity using BLASTn [11] and post-clustering curation was conducting using LULU [25] with default parameters to produce the final ASV table. Decipher [80] was used for ASV alignment, prior to construction of a phylogenetic tree using a GTR model with the R-package phagorn [72]. Singletons, spurious ASV with a mean relative abundance below 1E^−5^, or those assigned to the order Chloroplast, or the family Mitochondria were filtered from the dataset prior to statistical analysis. Phyloseq [52] and vegan [58] were used for all data transformations and calculation of ecological indices.

Differential abundance (DA) testing was done using DESeq2 [46] to identify DA by tissue, while controlling for dietary effects. Tissue-specific dietary effects on microbiota abundance were identified through pairwise comparison of dietary treatments to the control group, within each tissue. All DA testing was conducted with a significance threshold of FDR-corrected *p* ≤ 0.05 and absolute log2-fold change ≥ 1.0, following fold-change shrinkage according to apeglm [85, 86].

Metagenomic functional potential of microbiota were inferred as KEGG orthologs (KO), KEGG Enzyme Commission codes (EC) and MetaCyc metabolic pathways using PICRUSt2. Microbiota functions were plotted and analyzed using STAMP [59] to compare the functional potential of the microbes present on the three mucosal sites using a Kruskal Wallis H test followed by a Tukey–Kramer post-hoc test. Functions were considered significantly different when FDR corrected *p*-values were less than 0.01 and effect size was greater than 0.5.

Network reconstruction was conducted on tissue specific datasets (gut, gill, and skin) to evaluate microbial co-association patterns by mucosal site and identify site-specific keystone taxa. To reduce sparsity, individual datasets were filtered to remove ASV that accounted for less than 0.0001% relative abundance. Compositional data transformation and inference of sparse inverse covariance networks was conducted using SPIEC-EASI [40]. Network centrality metrics were tested for accuracy in describing network structure using the R package CINNA.

### RNA extraction and gene expression analysis

Gut, gill, and skin tissue, as well as PBL, were thawed and removed from RNALater. RNA was isolated using the RNeasy 96 HT RNA Isolation Kit (Qiagen) automated on the QiacubeHT. Concentrations and quality of RNA were assessed by NanoDrop 2000 (260/280 ≥ 1.8 and 260/230 ≥ 1.5). Samples below the acceptable thresholds were cleaned using a GeneJet RNA Clean-up Kit (Thermo Fisher Scientific). Twelve samples of each tissue were randomly selected for analysis on an RNA 6000 Nano chip (Agilent Technologies) to confirm RNA integrity (RIN ≥ 7.2). Removal of gDNA and reverse transcription were conducted in triplicate 20 µL reactions with 1000 ng of input RNA each using the iScript gDNA Clear cDNA Synthesis Kit (Bio-Rad Laboratories; Hercules, CA, USA) on a T100 thermocycler (Bio-Rad Laboratories). Primers for RT-qPCR analysis were taken from previously published literature where possible [5, 56, 70, 73, 74, 83], after confirming specificity in-silico, while other primers were designed using NCBI accessions and Primer-BLAST [81] (Additional file 2: Table S7). Primer sets were validated by running products on a 2% agarose gel to confirm specificity and size of intended target. Efficiency of each primer set was estimated on each tissue using duplicate six series five-fold serial dilutions of pooled sample cDNA standards. Each assay was conducted in duplicate 10 µL reactions with 1 µL of neat cDNA, 300 nM forward and reverse primer, and SsoAdvanced Universal Sybr Green Supermix (Bio-Rad Laboratories). The qPCR assays were run on a CFX96 (Bio-Rad Laboratories) for 35 cycles following recommended cycling parameters (Ta = 60 °C). Melt curve analysis was conducted to verify assay specificity.

Elongation factor 1 alpha (EF-1$\infty$), hypoxanthine-guanin phosphoribosyl transferase 1 (HPRT1), and RNA polymerase 2 subunit (RPABC2) served as reference genes with stability confirmed by the reference gene selection tool onboard the CFX96 (Bio-Rad Laboratories). Raw Ct values were efficiency corrected and normalized against the geometric mean of the reference genes using soft normalization priors in MCMC.qPCR [51]. Gene expression data were analyzed separately with a set of systemic-adaptive-immunity related genes assayed in the gut, skin, gill and PBL samples, and another set of mucosal-innate-immunity related genes analyzed across the three mucosal tissues only. The two gene sets were analyzed separately following the same procedures. Efficiency corrected Ct values for all genes were modeled using fixed effects of tissue, diet, and tissue-diet interaction, while controlling for random effects of tank and individual sample under a single Bayesian model. Outlier detection was done using the full model, with samples two standard deviations from the global sample-mean removed. Four samples were dropped from the systemic-adaptive gene set (1 gut, 1 PBL, and 2 skin samples) and three samples were dropped from the mucosal-innate gene set (1 gut and 2 skin samples). Gene-wise *p*-values from contrasts of interest were extracted from the model and adjusted for multiple comparison (FDR adjusted, *p* ≤ 0.05).

### Sea lice challenge

Following the diet trial, five fish per tank were transferred to common-garden tanks in a recirculating aquaculture system for challenge with sea lice (Salmon louse, *Lepeophtheirus salmonis*). Three common garden challenges were conducted with fish from multiple tanks of each dietary treatment equally represented in each challenge (40 fish challenge^−1^). Challenges followed the protocol outlined in Peterson et al. [62]. Briefly, a static bath challenge was conducted at a density of 100 infective copepods fish^−1^, with water supply returned after 4 h. Infections lasted 10–14 days prior to counting of infective sea lice and calculating right-side lice density [27]. In addition, surface-area (cm^2^) was estimated using the formula provided by Frederick et al. [23] to calculate lice surface-area^−1^. Lice density and lice surface-area^−1^ were tested for differences due to dietary treatment by one-way ANOVA, while controlling for diet-trial tank and sea-lice challenge replicate.

## Supplementary Information

**Additional file 1**: **Figure S1**. Water temperatures observed in the flow-through seawater rearing system throughout the trial; The **Figure S2**. Relative abundance of bacteria detected in positive and negative internal microbiota controls. ZymoBIOMICS® Microbial Community Standard (Zymo Research) was included at the DNA extraction step of the workflow to measure phylogenetic coverage and quantitative accuracy (A). On-plate no-template amplification negative controls were included with each PCR1 plate and yield very low concentration libraries (2,963 ± 1,462 reads; mean ± SD) (B); **Figure S3**. Rarefaction curve of species richness from samples collected from mucosal tissues of Atlantic salmon;**Figure S4**. Microbiota composition by sample type. Phylum level microbiota composition across dietary treatment are listed for the skin (A), gill (B), and gut (C) mucosa of Atlantic salmon, as well as the environmental samples (water and diet) (D). An upset plot (E) shows the total number of ASV observed by sample type as well as the overlap (Shared ASV) between sample types; **Figure S5**. Venn diagram showing overlap in differentially abundant microbiota ASV across pairwise tissue comparisons

**Additional file 2**: **Table S1**. Top 50 keystone gut microbiota identified through network analysis; **Table S2**. Top 50 keystone gill microbiota identified through network analysis; **Table S3**. Top 50 keystone skin microbiota identified through network analysis; **Table S4**. Differentially abundant inferred metagenomic KEGG Orthologs (KO) across mucosal tissues; **Table S5**. Differentially abundant inferred metagenomic KEGG Enzyme Commission (EC) codes across mucosal tissues; **Table S6**. Differentially abundant inferred metagenomic MetaCyc pathways across mucosal tissues; **Table S7**. Primer sequences used for RT-qPCR. Primers with listed references were taken from previously published literature, after confirming specificity in-silico, and all other primers were designed using NCBI Primer-BLAST with the listed accession as the target. NCBI accessions are taken from RefSeq where possible, with those accessions denoted by * coming from GenBank.

## Data Availability

Raw 16S rRNA gene sequencing data are publicly available on the NCBI repository (https://www.ncbi.nlm.nih.gov) under BioProject PRJNA663352. Notebooks containing the R code used for all data processing and analysis can be found on GitHub (https://github.com/jbledsoe/Salsal_TissueMicrobiota).

## Abbreviations

ASV: Amplicon sequence variant
DA: Differential abundance
KO: KEGG orthologs
MOS: Mannan-oligosaccharides
PBL: Peripheral blood leukocytes
PCA: Principal components analysis
PCoA: Principal coordinates analysis
PERMANOVA: Permutational multivariate analysis of variance
FDR: False discovery rate corrected *p*-value

## References

[1] Adams A (2019). Progress, challenges and opportunities in fish vaccine development. Fish Shellfish Immunol.

[2] Ángeles Esteban M. An overview of the immunological defenses in fish skin. ISRN Immunol 2012.

[3] Ashtiani M, Mirzaie M, Jafari M (2019). CINNA: an R/CRAN package to decipher central informative nodes in network analysis. Bioinformatics.

[4] Attia YA, Al-Harthi MA, Abo El-Maaty HM (2020). The effects of different oil sources on performance, digestive enzymes, carcass traits, biochemical, immunological, antioxidant, and morphometric responses of broiler chicks. Front Vet Sci.

[5] Austbø L, Aas IB, König M, Weli SC, Syed M, Falk K, Koppang EO (2014). Transcriptional response of immune genes in gills and the interbranchial lymphoid tissue of Atlantic salmon challenged with infectious salmon anemia virus. Dev Comp Immunol.

[6] Bjørndal T, Tusvik A. Economic analysis of on-growing of salmon post-smolts. Aquac Econ Manag 2020;1–32.

[7] Blaufuss PC, Bledsoe JW, Gaylord TG, Sealey WM, Overturf KE, Powell MS (2020). Selectionon a plant-based diet reveals changes in oral tolerance, microbiota and growth in rainbow trout (Oncorhynchusmykiss) when fed a high soy diet. Aquaculture.

[8] Bledsoe JW, Peterson BC, Swanson KS, Small BC (2016). Ontogenetic characterization of the intestinal microbiota of channel catfish through 16S rRNA gene sequencing reveals insights on temporal shifts and the influence of environmental microbes. PLoS ONE.

[9] Butt RL, Volkoff H (2019). Gut microbiota and energy homeostasis in fish. Front Endocrinol.

[10] Callahan BJ, McMurdie PJ, Rosen MJ, Han AW, Johnson AJA, Holmes SP (2016). DADA2: high- resolution sample inference from Illumina amplicon data. Nat Methods.

[11] Camacho C, Coulouris G, Avagyan V, Ma N, Papadopoulos J, Bealer K, Madden TL (2009). BLAST+: architecture and applications. BMC Bioinformat.

[12] Cheaib B, Yang P, Kazlauskaite R, Lindsay E, Heys C, Dwyer T, DeNoa M, Shaal P, Sloan W, Ijaz UZ, Llewellyn MS (2021). Genome erosion and evidence for an intracellular niche-exploring the biology of mycoplasmas in Atlantic salmon. Aquaculture.

[13] Chiarello M, Auguet J-C, Bettarel Y, Bouvier C, Claverie T, Graham NA, Rieuvilleneuve F, Sucré E, Bouvier T, Villéger S (2018). Skin microbiome of coral reef fish is highly variable and driven by host phylogeny and diet. Microbiome.

[14] Choteau L, Parny M, Francois N, Bertin B, Fumery M, Dubuquoy L, Takahashi K, Colombel J-F, Jouault T, Poulain D (2016). Role of mannose-binding lectin in intestinal homeostasis and fungal elimination. Mucosal Immunol.

[15] Crippen TL, Bootland LM, Leong J-AC, Fitzpatrick MS, Schreck CB, Vella AT (2001). Analysis of salmonid leukocytes purified by hypotonic lysis of erythrocytes. J Aquat Anim Health.

[16] De Schryver P, Vadstein O (2014). Ecological theory as a foundation to control pathogenic invasion in aquaculture. ISME J.

[17] Dehler CE, Secombes CJ, Martin SA (2017). Environmental and physiological factors shape the gut microbiota of Atlantic salmon parr (Salmo salar L.). Aquaculture.

[18] Dimitroglou A, Merrifield DL, Spring P, Sweetman J, Moate R, Davies SJ (2010). Effects of mannan oligosaccharide (MOS) supplementation on growth performance, feed utilization, intestinal histology and gut microbiota of gilthead sea bream (*Sparus aurata*). Aquaculture.

[19] Dimitroglou A, Merrifield DL, Moate R, Davies SJ, Spring P, Sweetman J, Bradley G (2009). Dietary mannan oligosaccharide supplementation modulates intestinal microbial ecology and improves gut morphology of rainbow trout, *Oncorhynchus mykiss (Walbaum)1*. J Anim Sci.

[20] Douglas GM, Maffei VJ, Zaneveld JR, Yurgel SN, Brown JR, Taylor CM, Huttenhower C, Langille MG (2020). PICRUSt2 for prediction of metagenome functions. Nat Biotechnol.

[21] Fadrosh DW, Ma B, Gajer P, Sengamalay N, Ott S, Brotman RM, Ravel J (2014). An improved dual- indexing approach for multiplexed 16S rRNA gene sequencing on the Illumina MiSeq platform. Microbiome.

[22] Fast MD, Sims DE, Burka JF, Mustafa A, Ross NW (2002). Skin morphology and humoral non-specific defense parameters of mucus and plasma in rainbow trout, coho and Atlantic salmon. Comp Biochem Physiol A Mol Integr Physiol.

[23] Frederick C, Brady DC, Bricknell I (2017). Landing strips: model development for estimating body surface area of farmed Atlantic salmon (*Salmo salar*). Aquaculture.

[24] Friberg IM, Taylor JD, Jackson JA (2019). Diet in the driving seat: natural diet-immunity-microbiome interactions in wild fish. Front Immunol.

[25] Frøslev TG, Kjøller R, Bruun HH, Ejrnæs R, Brunbjerg AK, Pietroni C, Hansen AJ (2017). Algorithm for post-clustering curation of DNA amplicon data yields reliable biodiversity estimates. Nat Commun.

[26] Gajardo K, Jaramillo-Torres A, Kortner TM, Merrifield DL, Tinsley J, Bakke AM, Krogdahl Å (2017). Alternative protein sources in the diet modulate microbiota and functionality in the distal intestine of Atlantic salmon (*Salmo salar*). Appl Environ Microbiol.

[27] Gjerde B, Ødegård J, Thorland I (2011). Estimates of genetic variation in the susceptibility of Atlantic salmon (*Salmo salar*) to the salmon louse *Lepeophtheirus salmonis*. Aquaculture.

[28] Gomez D, Sunyer JO, Salinas I (2013). The mucosal immune system of fish: the evolution of tolerating commensals while fighting pathogens. Fish Shellfish Immunol.

[29] Gonçalves A, Gallardo-Escárate C (2017). Microbiome dynamic modulation through functional diets based on pre-and probiotics (mannan-oligosaccharides and *Saccharomyces cerevisiae*) in juvenile rainbow trout (Oncorhynchus mykiss). J Appl Microbiol.

[30] Guerreiro I, Oliva-Teles A, Enes P (2018). Prebiotics as functional ingredients: focus on Mediterranean fish aquaculture. Rev Aquac.

[31] Hervé M, Hervé MM. Package ‘RVAideMemoire’ 2020. See https://CRAN.R-project.RVAideMemoire.

[32] Hirazawa N, Oshima SI, Hara T, Mitsuboshi T, Hata K (2001). Antiparasitic effect of medium-chain fatty acids against the ciliate Cryptocaryon irritans infestation in the red sea bream Pagrus major. Aquaculture.

[33] Hu Y, Maisey K, Subramani PA, Liu F, Flores-Kossack C, Imarai M, Secombes CJ, Wang T (2018). Characterization of rainbow trout peripheral blood leucocytes prepared by hypotonic lysis of erythrocytes, and analysis of their phagocytic activity, proliferation and response to PAMPs and proinflammatory cytokines. Dev Comp Immunol.

[34] Huang Q, Sha RC, Deng Y, Mao Y, Wang C, Zhang T, Leung KMY (2020). Diversity of gut microbiomes in marine fishes is shaped by host-related factors. Mol Ecol.

[35] Huang CB, Alimova Y, Myers TM, Ebersole JL (2011). Short-and medium-chain fatty acids exhibit antimicrobial activity for oral microorganisms. Arch Oral Biol.

[36] Johnston CE, Horney BS, Deluca S, MacKenzie A, Eales JG, Angus R (1994). Changes in alkaline phosphatase isoenzyme activity in tissues and plasma of Atlantic salmon (*Salmo salar*) before and during smoltification and gonadal maturation. Fish Physiol Biochem.

[37] Klindworth A, Pruesse E, Schweer T, Peplies J, Quast C, Horn M, Glöckner FO (2013). Evaluation of general 16S ribosomal RNA gene PCR primers for classical and next-generation sequencing-based diversity studies. Nucleic Acids Res.

[38] Kononova SV, Zinchenko DV, Muranova TA, Belova NA, Miroshnikov AI (2019). Intestinal microbiota of salmonids and its changes upon introduction of soy proteins to fish feed. Aquacult Int.

[39] Koppang EO, Kvellestad A, Fischer U. Fish mucosal immunity: gill, Mucosal Health in Aquaculture. Elsevier 2015;93–133.

[40] Kurtz ZD, Müller CL, Miraldi ER, Littman DR, Blaser MJ, Bonneau RA (2015). Sparse and compositionally robust inference of microbial ecological networks. PLoS Comput Biol.

[41] Lallès JP (2020). Intestinal alkaline phosphatase in the gastrointestinal tract of fish: biology, ontogeny, and environmental and nutritional modulation. Rev Aquac.

[42] Leary SL, Underwood W, Anthony R, Cartner S, Corey D, Grandin T, Greenacre C, Gwaltney-Brant S, McCrackin M, Meyer R. AVMA guidelines for the euthanasia of animals: 2013 edition. American Veterinary Medical Association Schaumburg, IL 2013.

[43] Leclercq E, Pontefract N, Rawling M, Valdenegro V, Aasum E, Andujar LV, Migaud H, Castex M, Merrifield D (2020). Dietary supplementation with a specific mannan-rich yeast parietal fraction enhances the gut and skin mucosal barriers of Atlantic salmon (*Salmo salar*) and reduces its susceptibility to sea lice (*Lepeophtheirus salmonis*). Aquaculture.

[44] Legrand TP, Wynne JW, Weyrich LS, Oxley AP (2020). A microbial sea of possibilities: current knowledge and prospects for an improved understanding of the fish microbiome. Rev Aquac.

[45] Legrand TP, Catalano SR, Wos-Oxley ML, Stephens F, Landos M, Bansemer MS, Stone DA, Qin JG, Oxley A (2018). The inner workings of the outer surface: skin and gill microbiota as indicators of changing gut health in yellowtail kingfish. Front Microbiol.

[46] Love MI, Huber W, Anders S (2014). Moderated estimation of fold change and dispersion for RNA-seq data with DESeq2. Genome Biol.

[47] Løvoll M, Johnsen H, Boshra H, Bøgwald J, Sunyer JO, Dalmo RA (2007). The ontogeny and extrahepatic expression of complement factor C3 in Atlantic salmon (*Salmo salar*). Fish Shellfish Immunol.

[48] Luo L, Xue M, Vachot C, Geurden I, Kaushik S (2014). Dietary medium chain fatty acids from coconut oil have little effects on postprandial plasma metabolite profiles in rainbow trout (*Oncorhynchus mykiss*). Aquaculture.

[49] Mandal S, Van Treuren W, White RA, Eggesbø M, Knight R, Peddada SD (2015). Analysis of composition of microbiomes: a novel method for studying microbial composition. Microb Ecol Health Dis.

[50] Martin SA, Król E (2017). Nutrigenomics and immune function in fish: new insights from omics technologies. Dev Comp Immunol.

[51] Matz MV, Wright RM, Scott JG (2013). No control genes required: Bayesian analysis of qRT-PCR data. PLoS ONE.

[52] McMurdie PJ, Holmes S (2013). phyloseq: an R package for reproducible interactive analysis and graphics of microbiome census data. PLoS ONE.

[53] Micallef G, Cash P, Fernandes JM, Rajan B, Tinsley JW, Bickerdike R, Martin SA, Bowman AS (2017). Dietary yeast cell wall extract alters the proteome of the skin mucous barrier in Atlantic Salmon (*Salmo salar*): increased abundance and expression of a calreticulin-like protein. PLoS ONE.

[54] Minich JJ, Sanders JG, Amir A, Humphrey G, Gilbert JA, Knight R (2019). Quantifying and understanding well-to-well contamination in microbiome research. mSystems..

[55] Minich JJ, Poore GD, Jantawongsri K, Johnston C, Bowie K, Bowman J, Knight R, Nowak B, Allen EE (2020). Microbial ecology of Atlantic salmon (*Salmo salar*) hatcheries: impacts of the built environment on fish mucosal microbiota. Appl Environ Microbiol.

[56] Mutoloki S, Cooper GA, Marjara IS, Koop BF, Evensen Ø (2010). High gene expression of inflammatory markers and IL-17A correlates with severity of injection site reactions of Atlantic salmon vaccinated with oil-adjuvanted vaccines. BMC Genom.

[57] Naylor RL, Hardy RW, Bureau DP, Chiu A, Elliott M, Farrell AP, Forster I, Gatlin DM, Goldburg RJ, Hua K (2009). Feeding aquaculture in an era of finite resources. PNAS.

[58] Oksanen J, Blanchet FG, Kindt R, Legendre P, Minchin PR, O’hara R, Simpson GL, Solymos P, Stevens MHH, Wagner H. Package ‘vegan’. Community ecology package, version. 2, 2013:1–295.

[59] Parks DH, Tyson GW, Hugenholtz P, Beiko RG (2014). STAMP: statistical analysis of taxonomic and functional profiles. Bioinformatics.

[60] Perry WB, Lindsay E, Payne CJ, Brodie C, Kazlauskaite R (2020). The role of the gut microbiome in sustainable teleost aquaculture. Proc R Soc B.

[61] Peterson BC, Peatman E, Ourth D, Waldbieser G (2015). Effects of a phytogenic feed additive on growth performance, susceptibility of channel catfish to *Edwardsiella ictaluri* and levels of mannose binding lectin. Fish Shellfish Immunol.

[62] Peterson BC, Burr GS, Pietrak MR, Proestou DA (2020). Genetic Improvement of North American Atlantic Salmon and the Eastern Oyster *Crassostrea virginica* at the US Department of Agriculture-Agricultural Research Service National Cold Water Marine Aquaculture Center. N Am J Aquac.

[63] Priya S, Burns MB, Ward T, Mars RA, Adamowicz B, Lock EF, Kashyap PC, Knights D, Blekhman R (2021). Shared and disease-specific host gene-microbiome interactions across human diseases. BioRxiv.

[64] Quast C, Pruesse E, Yilmaz P, Gerken J, Schweer T, Yarza P, Peplies J, Glöckner FO (2012). The SILVA ribosomal RNA gene database project: improved data processing and web-based tools. Nucleic Acids Res.

[65] Rasmussen JA, Villumsen KR, Ernst M, Hansen M, Forberg T, Gopalakrishnan S, Gilbert MTP, Boejesen AM, Kristiansen K, Limborg MT (2022). A multi-omics approach unravels metagenomic and metabolic alterations of a probiotic and synbiotic additive in rainbow trout (*Oncorhynchus mykiss*). Microbiome.

[66] Rasmussen JA, Villumsen KR, Duchêne DA, Puetz LC, Delmont TO, Sveier H, von Gersdorff Jøregensen L, Præbel K, Martin MD, Bojesen AM, Gilbert MTP (2021). Genome-resolved metagenomics suggests a mutualistic relationship between *Mycoplasma* and salmonid hosts. Commun Biol.

[67] Refstie S, Baeverfjord G, Seim RR, Elvebø O (2010). Effects of dietary yeast cell wall β-glucans and MOS on performance, gut health, and salmon lice resistance in Atlantic salmon (*Salmo salar*) fed sunflower and soybean meal. Aquaculture.

[68] Rodriguez-Estrada U, Satoh S, Haga Y, Fushimi H, Sweetman J (2013). Effects of inactivated Enterococcus faecalis and mannan oligosaccharide and their combination on growth, immunity, and disease protection in rainbow trout. N Am J Aquac.

[69] Rombout JH, Yang G, Kiron V (2014). Adaptive immune responses at mucosal surfaces of teleost fish. Fish Shellfish Immunol.

[70] Sahlmann C, Sutherland BJ, Kortner TM, Koop BF, Krogdahl Å, Bakke AM (2013). Early response of gene expression in the distal intestine of Atlantic salmon (*Salmo salar L*.) during the development of soybean meal induced enteritis. Fish Shellfish Immunol.

[71] Salinas I, Zhang Y-A, Sunyer JO (2011). Mucosal immunoglobulins and B cells of teleost fish. Dev Comp Immunol.

[72] Schliep KP (2011). phangorn: phylogenetic analysis in R. Bioinformatics.

[73] Skugor S, Glover KA, Nilsen F, Krasnov A (2008). Local and systemic gene expression responses of Atlantic salmon (*Salmo salar* L.) to infection with the salmon louse (*Lepeophtheirus salmonis*). BMC Genom.

[74] Tadiso TM, Krasnov A, Skugor S, Afanasyev S, Hordvik I, Nilsen F (2011). Gene expression analyses of immune responses in Atlantic salmon during early stages of infection by salmon louse (*Lepeophtheirus salmonis*) revealed bi-phasic responses coinciding with the copepod-chalimus transition. BMC Genom.

[75] Tocher DR (2003). Metabolism and functions of lipids and fatty acids in teleost fish. Rev Fish Sci.

[76] Torrecillas S, Montero D, Izquierdo M (2014). Improved health and growth of fish fed mannan oligosaccharides: potential mode of action. Fish Shellfish Immunol.

[77] Torrecillas S, Montero D, Caballero MJ, Pittman KA, Custódio M, Campo A, Sweetman J, Izquierdo M (2015). Dietary Mannan Oligosaccharides: Counteracting the Side Effects of Soybean Meal Oil Inclusion on European Sea Bass (*Dicentrarchus labrax)* Gut Health and Skin Mucosa Mucus Production?. Front Immunol.

[78] Torrecillas S, Rivero-Ramírez F, Izquierdo M, Caballero M, Makol A, Suarez-Bregua P, Fernández- Montero A, Rotllant J, Montero D (2018). Feeding European Sea bass (*Dicentrarchus labrax*) juveniles with a functional synbiotic additive (mannan oligosaccharides and *Pediococcus acidilactici*): An effective tool to reduce low fishmeal and fish oil gut health effects?. Fish Shellfish Immunol.

[79] van Veelen HPJ, Falcão Salles J, Matson KD, van der Velde M, Tieleman BI (2020). Microbial environment shapes immune function and cloacal microbiota dynamics in zebra finches *Taeniopygia guttata*. Animal Microbiome.

[80] Wright ES. Using DECIPHER v2. 0 to analyze big biological sequence data in R. R Journal 2016:8.

[81] Ye J, Coulouris G, Zaretskaya I, Cutcutache I, Rozen S, Madden TL (2012). Primer-BLAST: a tool to design target-specific primers for polymerase chain reaction. BMC Bioinf.

[82] Zhang Y-A, Salinas I, Li J, Parra D, Bjork S, Xu Z, LaPatra SE, Bartholomew J, Sunyer JO (2010). IgT, a primitive immunoglobulin class specialized in mucosal immunity. Nat Immunol.

[83] Zhang Z, Chi H, Niu C, Bøgwald J, Dalmo RA (2011). Molecular cloning and characterization of Foxp3 in Atlantic salmon (*Salmo salar*). Fish Shellfish Immunol.

[84] Zhao H, Li C, Beck BH, Zhang R, Thongda W, Davis DA, Peatman E (2015). Impact of feed additives on surface mucosal health and columnaris susceptibility in channel catfish fingerlings, *Ictalurus punctatus*. Fish & Shellfish Immunol.

[85] Zhu A, Ibrahim JG, Love MI (2019). Heavy-tailed prior distributions for sequence count data: removing the noise and preserving large differences. Bioinformatics.

[86] Zhu Q, Gao R, Zhang Y, Pan D, Zhu Y, Zhang X, Yang R, Jiang R, Xu Y, Qin H (2018). Dysbiosis signatures of gut microbiota in coronary artery disease. Physiol Genom.

