# Supplementary material for: Functional feeds marginally alter immune expression and microbiota of Atlantic salmon (Salmo salar) gut, gill, and skin mucosa though evidence of tissue-specific signatures and host–microbe coadaptation remain
Source: Anim Microbiome. 2022 Mar 10;4:20. doi: 10.1186/s42523-022-00173-0 (PMC8908560; doi:10.1186/s42523-022-00173-0)
Supplement: Supplementary file 1 — Additional file 1: Figure S1. Water temperatures observed in the flow-through seawater rearing system throughout the trial; The Figure S2. Relative abundance of bacteria detected in positive and negative internal microbiota controls. ZymoBIOMICS® Microbial Community Standard (Zymo Research) was included at the DNA extraction step of the workflow to measure phylogenetic coverage and quantitative accuracy (A). On-plate no-template amplification negative controls were included with each PCR1 plate and yield very low concentration libraries (2,963 ± 1,462 reads; mean ± SD) (B); Figure S3. Rarefaction curve of species richness from samples collected from mucosal tissues of Atlantic salmon;Figure S4. Microbiota composition by sample type. Phylum level microbiota composition across dietary treatment are listed for the skin (A), gill (B), and gut (C) mucosa of Atlantic salmon, as well as the environmental samples (water and diet) (D). An upset plot (E) shows the total number of ASV observed by sample type as well as the overlap (Shared ASV) between sample types; Figure S5. Venn diagram showing overlap in differentially abundant microbiota ASV across pairwise tissue comparisons [file 42523_2022_173_MOESM1_ESM.pdf]

Symmetry in immune expression and microbiota of Atlantic salmon (*Salmo salar*) gill, gut, and skin reveal host-microbe coadaptations that are marginally perturbed by functional feeds

Additional File 1

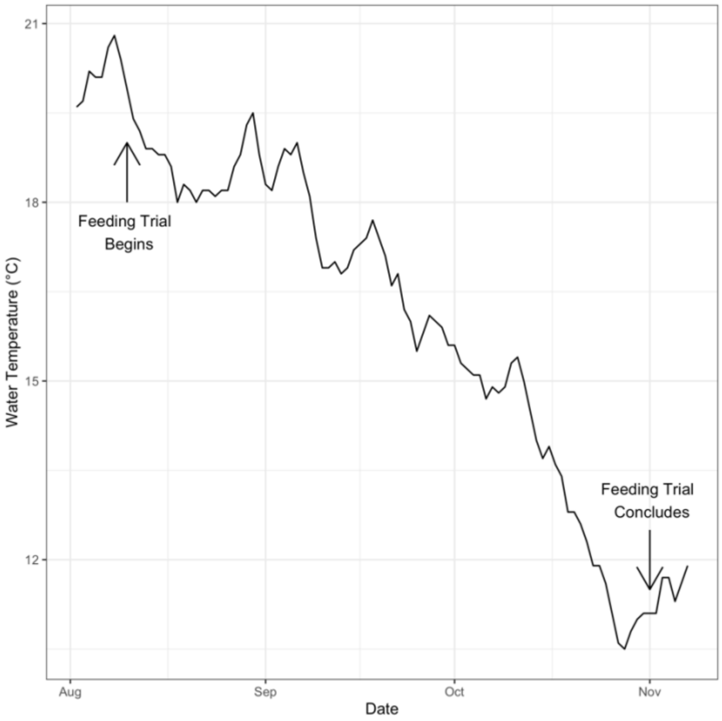

Figure S1. Water temperatures observed in the flow-through seawater rearing system throughout the trial.

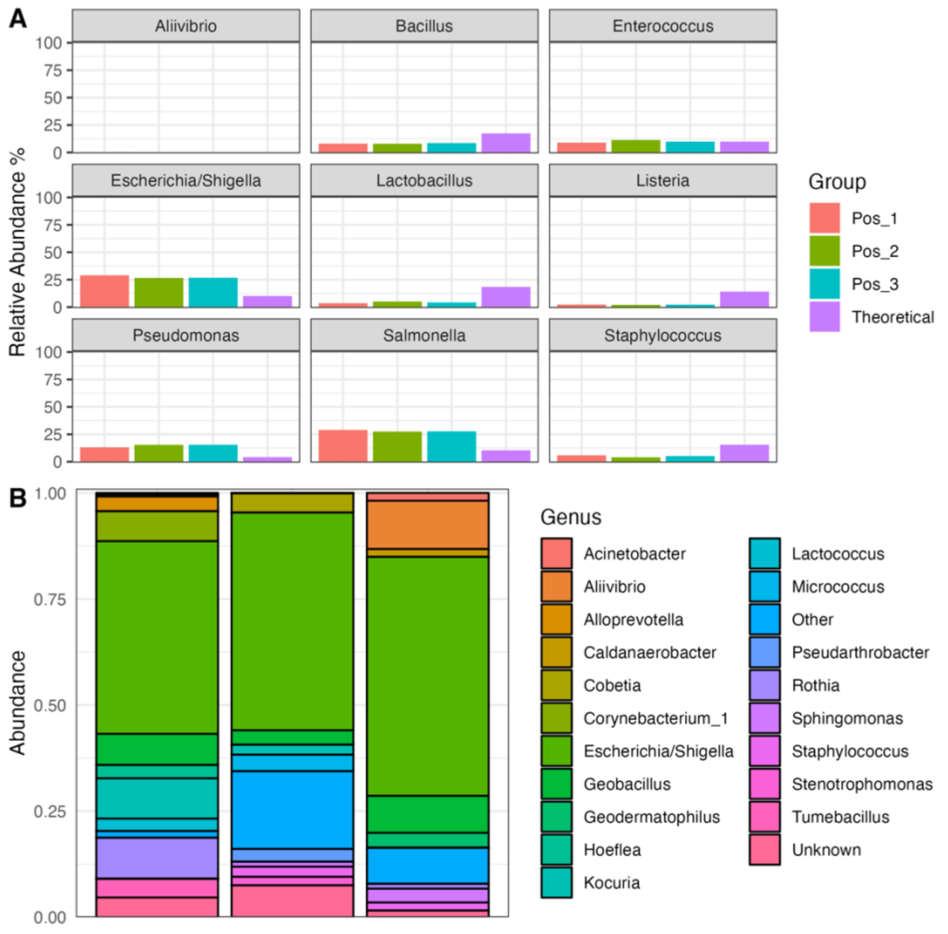

Figure S2. Relative abundance of bacteria detected in positive and negative internal microbiota controls. ZymoBIOMICS® Microbial Community Standard (Zymo Research) positive controls (A) were included at the DNA extraction step of theworkflow to measure phylogenetic coverage and quantitative accuracy. A few reads in two of the positive controls were assigned to the genus *Allivibrio* (> 0.001% relative abundance) which was not included in the positive community, but was highly abundant among experimental samples. On-plate no-template negative controls (B) were included with each PCR1 plate and yielded very low concentration libraries (2,963 ± 1,462 reads; mean ± SD).

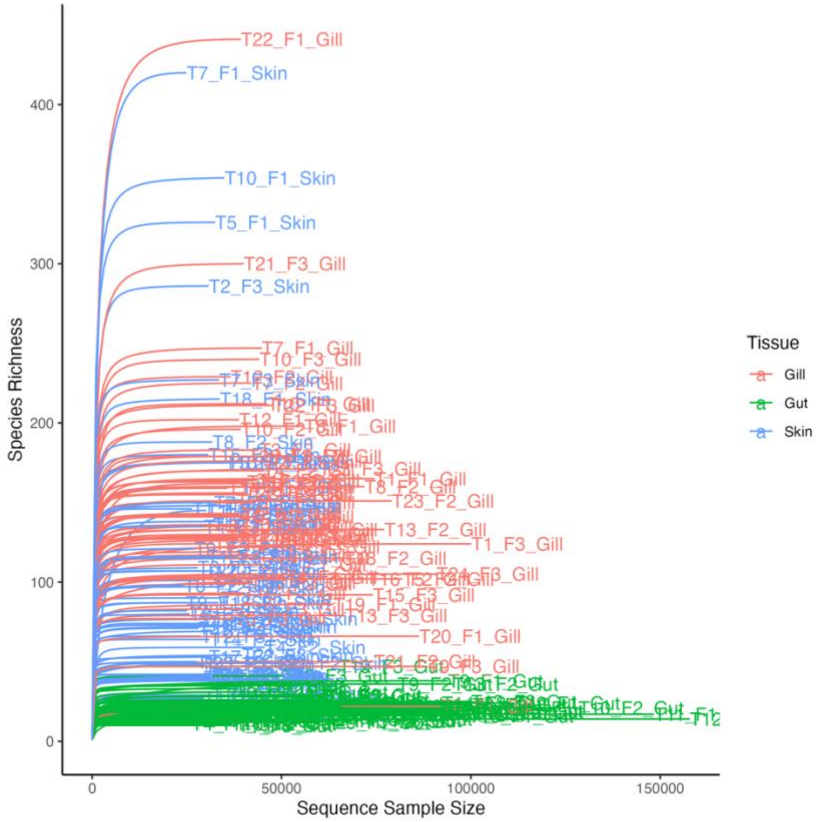

Figure S3. Rarefaction curves for species richness from samples collected from mucosal tissues of Atlantic salmon.

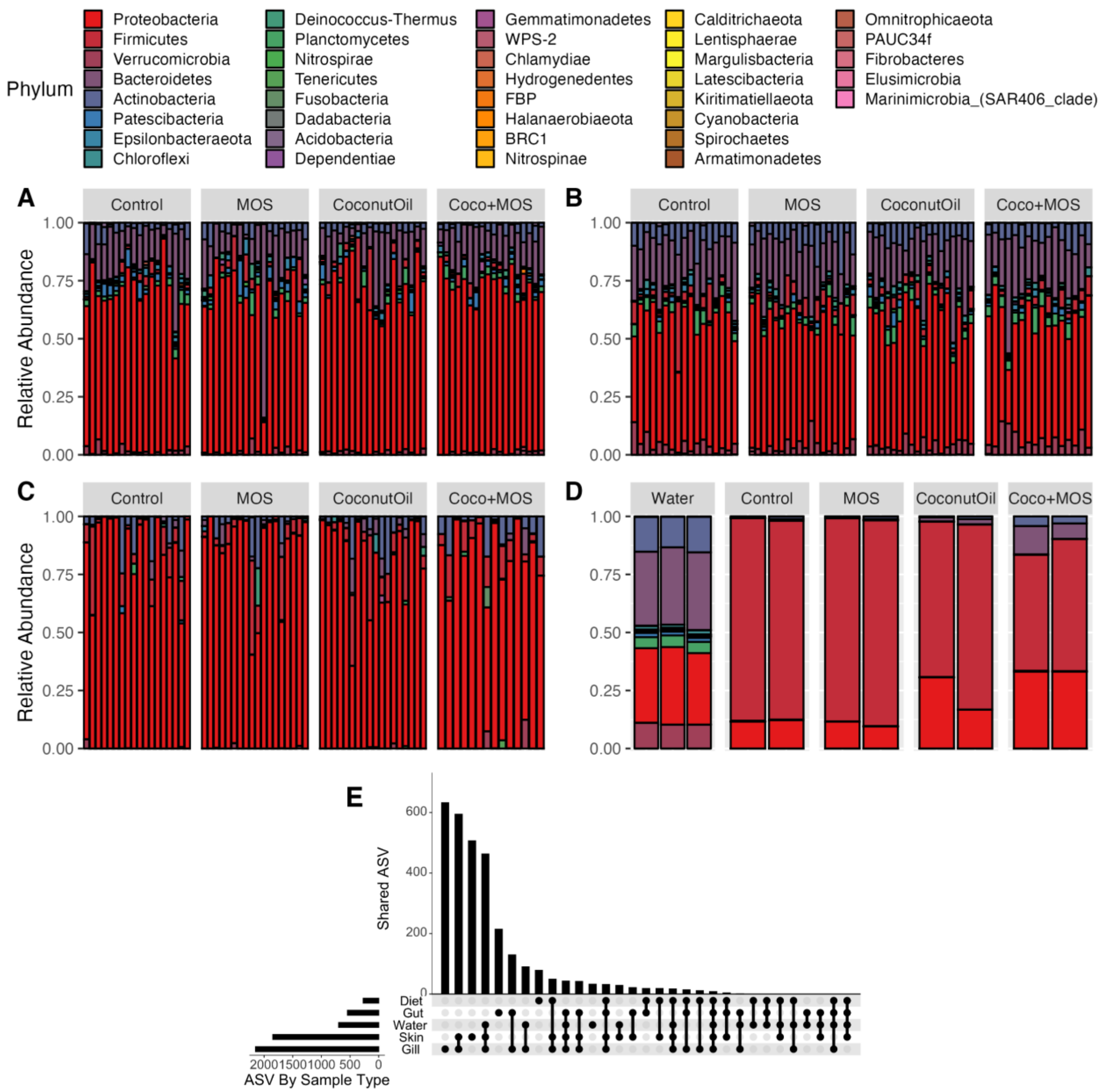

Figure S4. Microbiota composition by sample type. Phylum level microbiota composition across dietary treatment are shown for the skin (A), gill (B), and gut (C) mucosa of Atlantic salmon, as well as the environmental samples (water and diet) (D). An upset plot (E) shows the total number of ASV observed by sample type and the overlap (Shared ASV) between sample types.

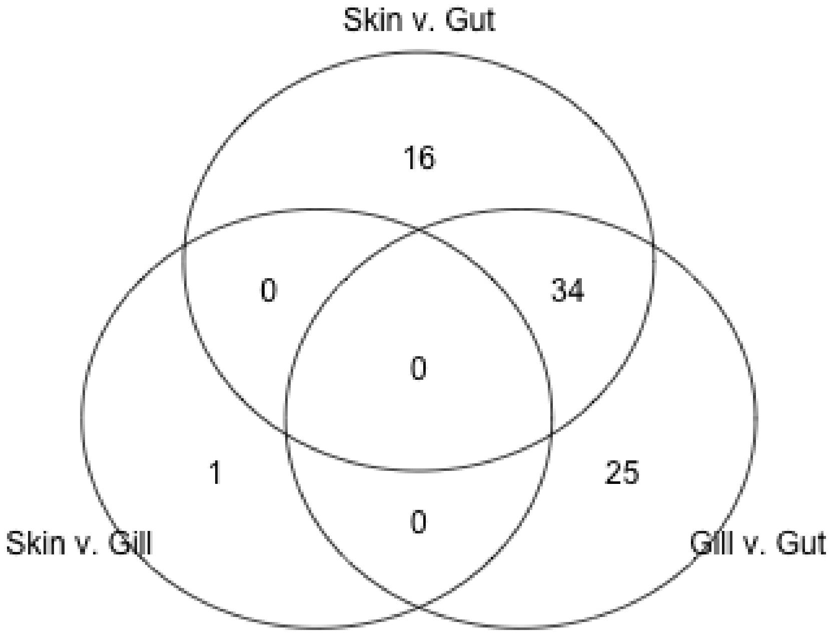

Figure S5. Venn diagram showing overlap in differentially abundant microbiota (ASV) across pairwise tissue comparisons.
